# Injury Rates, Mechanisms, Risk Factors and Prevention Strategies in Youth Rugby Union: What’s All the Ruck-Us About? A Systematic Review and Meta-analysis

**DOI:** 10.1007/s40279-023-01826-z

**Published:** 2023-05-16

**Authors:** Stephen W. West, Isla J. Shill, Stuart Bailey, Reid A. Syrydiuk, K. Alix Hayden, Debbie Palmer, Amanda M. Black, Brent E. Hagel, Keith A. Stokes, Carolyn A. Emery

**Affiliations:** 1 Centre for Health, and Injury and Illness Prevention in Sport, University of Bath, Bath, UK; 2 UK Collaborating Centre on Injury & Illness Prevention in Sport (UKCCIIS), Edinburgh & Bath, UK; 3 Sport Injury Prevention Research Centre, Faculty of Kinesiology, University of Calgary, Calgary, Canada; 4 O’Brien Institute of Public Health, University of Calgary, Calgary, Canada; 5 Hotchkiss Brain Institute, University of Calgary, Calgary, Canada; 6 School of Applied Sciences, Edinburgh Napier University, Edinburgh, UK; 7 Alberta Children’s Hospital Research Institute, University of Calgary, Calgary, Canada; 8 Michigan Concussion Center, School of Kinesiology, University of Michigan, Ann Arbor, USA; 9 Libraries and Cultural Resources, University of Calgary, Calgary, Canada; 10 Edinburgh Sports Medicine Research Network, Institute for Sport, PE and Health Sciences, University of Edinburgh, Edinburgh, UK; 11 Division of Rheumatology, Orthopaedics and Dermatology, School of Medicine, University of Nottingham, Nottingham, UK; 12 Human Performance Laboratory, Faculty of Kinesiology, University of Calgary, Calgary, Canada; 13 Department of Community Health Sciences, Cumming School of Medicine, University of Calgary, Calgary, Canada; 14 Rugby Football Union, Twickenham, London, UK; 15 Department of Pediatrics, Cumming School of Medicine, University of Calgary, Calgary, Canada

## Abstract

**Background:**

Rugby Union is a collision team sport played globally. Despite this, significant concerns have been raised regarding the sport’s safety, particularly in youth players. Given this, a review of injury rates, risk factors and prevention strategies is required across different youth age groups as well as in males and females.

**Objective:**

The objective of this systematic review (SR) and meta-analysis was to investigate injury and concussion rates, risk factors and primary prevention strategies in youth rugby.

**Methods:**

To be included, studies were required to report either rates, risk factors or prevention strategies in youth rugby and to have a randomised controlled trial, quasi-experimental, cohort, case control, or ecological study design. Exclusion criteria included non-peer-reviewed grey literature, conference abstracts, case studies, previous systematic reviews and studies not written in English. Nine databases were searched. The full search strategy and list of sources are available and pre-registered on PROSPERO (Ref: CRD42020208343). Each study was assessed for risk of bias using the Downs and Black quality assessment tool. Meta-analyses were conducted using a DerSimonian Laird random effect model for each age group and sex.

**Results:**

Sixty-nine studies were included in this SR. The match injury rates (using a 24-h time-loss definition) were 40.2/1000 match hours (95% CI 13.9–66.5) in males and 69.0/1000 match hours (95% CI 46.8–91.2) in females. Concussion rates were 6.2/1000 player-hours (95% CI 5.0–7.4) for males and 33.9/1000 player-hours (95% CI: 24.1–43.7) for females. The most common injury site was lower extremity (males) and the head/neck (females). The most common injury type was ligament sprain (males) and concussion (females). The tackle was the most common event associated with injury in matches (55% male, 71% females). Median time loss was 21 days for males and 17 days for females. Twenty-three risk factors were reported. The risk factors with the strongest evidence were higher levels of play and increasing age. Primary injury prevention strategies were the focus of only eight studies and included law changes (*n* = 2), equipment (*n* = 4), education (*n* = 1) and training (*n* = 1). The prevention strategy with the most promising evidence was neuromuscular training. The primary limitations included a broad range of injury definitions (*n* = 9) and rate denominators (*n* = 11) used, as well as a limited number of studies which could be included in the meta-analysis for females (*n* = 2).

**Conclusion:**

A focus on high-quality risk factor and primary prevention evaluation should be considered in future studies. Targeting primary prevention and stakeholder education remain key strategies in the prevention, recognition and management of injuries and concussions in youth rugby.

**Supplementary Information:**

The online version contains supplementary material available at 10.1007/s40279-023-01826-z.

## Key Points

**Table:** 

Injury and concussion rates in male and female youth rugby union players are high, and despite a paucity of studies focused on females, it appears the highest rates of both injury and concussion are reported in females.
The most common injury sites and injury type in youth rugby are lower extremity and ligament sprain in males, and head/ neck and concussion in females, with the tackle being the most common injurious event in matches (males- 55%, females- 71%).
Twenty-three risk factors were reported, with higher levels of play and increasing age displaying the strongest evidence for injury occurrence; however, no female studies examining risk factors were identified.
Only 8 studies were found to evaluate prevention strategies across law changes (*n* = 2), equipment (*n* = 4), education (*n* = 1) and training (*n* = 1). While neuromuscular training was the most promising strategy, there were no strategies evaluated in a female population.

## Introduction

Rugby Union is a collision team sport that is played by nearly 10 million people [1]. Despite the global popularity of the sport and its recent growth, there have been significant safety concerns regarding injury and concussion rates, particularly for youth. This was exemplified in 2019 by the banning of the sport (temporarily), in Nova Scotia, Canada [2]. Furthermore, there have been calls for a ban on tackling in the youth game in the United Kingdom [3]. In response to this call, Tucker et al. [4] highlighted that in the youth game, “neither the incidence nor severity of injury have been thoroughly identified and understood, and thus nor have the specific mechanisms and risk factors for injury”. This response also highlighted the key issues of differences in reporting of injury rates across youth rugby, making comparisons between studies challenging. Despite this, several previous reviews have focused on the risk of injury in youth rugby [5–7] and on concussion risk specifically [8, 9]. To date, however, these reviews have pooled estimates across ages 12–18 years [5], ≤ 18 years [9], 9–19 years [7], < 20 years [8] and < 21 years [6]. Furthermore, these studies have not stratified by sex. A review that stratifies both injury and concussion rates across specific age groups (e.g., U12, 12–14 years, 15–18 years) and across sexes was needed to offer greater insight than across all ages combined below 18 years. While these steps (establishing the rate of injury and the mechanisms of these injuries) are vital in the van Mechelen sequence of prevention [10], there are currently no reviews examining risk factors and primary prevention strategies in youth rugby. Given this, the objectives of this systematic review and meta-analysis are to investigate injury rates (and concussion rates specifically), types, mechanisms, risk factors and primary prevention strategies in youth rugby union.

## Methods

A systematic search and review of all youth-related rugby articles was undertaken. The review was reported and written in accordance with the Preferred Reporting Items for Systematic Reviews and Meta Analysis (PRISMA) statement [11] and was pre-registered on PROSPERO (record number: CRD42020208343). After initial data extraction was undertaken, and it was clear that enough studies presented information regarding rates, the protocol was refined to include a meta-analysis of injury and concussion rates separately for both male and female youth across three age groups.

### Data Sources and Searches

The search strategy was developed by an expert health sciences librarian (KAH). Nine databases were searched for this review: Ovid MEDLINE (R) In-Process & Other Non-Indexed Citations, CINAHL Plus with Full Text (Ebsco), APA PsycINFO (OVID), Cochrane Central Register of Controlled Trials (OVID), Cochrane Database of Systematic Reviews (OVID), SPORTDiscus with Full Text (Ebsco), EMBASE (OVID), ERIC (Ebsco) and the Web of Science Core Collection, which includes Science Citation Index—Expanded, Social Sciences Citation Index, Arts & Humanities Citation Index, Conference Proceedings Citation Index—Science, Conference Proceedings Citation Index—Social Sciences & Humanities and Emerging Sources Citation Index. No date limits were set with databases searched from the date of inception to the date of the search. The searches were originally conducted on September 21, 2020 and updated January 3, 2022. The search strategy included terms related to injury, youth and rugby (for the full search strategy, see Appendix 1 in the electronic supplementary material [ESM]). Search results were exported and uploaded into Covidence (Covidence systematic review software, Veritas Health Innovation, Melbourne, Australia) software for title/abstract and full-text screening. Manual searches of reference lists of previously cited systematic reviews as well as included articles were undertaken to capture any further studies not retrieved through the database search.

### Study Selection

All duplicate articles retrieved from online databases were removed automatically through Covidence. Prior to screening, a calibration exercise of 50 randomly selected titles/abstracts was conducted to ensure selection criteria were clear and applied in a similar way by all reviewers. Inter-rater reliability was measured using percentage agreement. Screening of titles and abstracts was undertaken by two authors (SWW, IJS), with disagreements discussed and resolved by a third author (CAE). To be included, the study must have reported at least one of the main outcomes of the review (i.e., rates, risk factors, primary prevention strategies). Study types included randomised controlled trials (RCTs), cluster RCTs, quasi-experimental studies, prospective/historical cohort studies, case–control studies, cross-sectional studies, case series and ecological studies. Only English papers were included. Cross-sectional studies were included in the initial screening; however, on review it was deemed that there was ample evidence using stronger cohort study designs for these to be removed. This was done given the potential for bias associated with cross-sectional study designs and was the case for 11 studies. Non-peer reviewed grey literature, conference abstracts and case studies were excluded. Studies with players aged 18 years or under playing in ‘U18’ rugby or defined as school-based rugby were included. If a study reported on players both ≥ 18 years and < 18 years of age then the study was included if data related to the U18 group could be extracted alone or if only a small number (< 5%) of participants were aged ≥ 18 years (13% of included studies). Previously published systematic reviews and meta-analyses were used to inform the review but were not included in the analysis. Full-text screening was completed by the same two authors (SWW, IJS) with disagreements resolved by CAE.

### Data Extraction and Quality Assessment

Prior to data extraction, a model extraction template was produced and tested by four authors (SWW, SB and IJS, RS) for three studies (one focusing on epidemiology of injury, one on risk factors and one on prevention strategies). This process led to refinement of the template for use on the included studies and also acted as a training opportunity for each data extractor. Four authors (two pairs [SWW, SB and IJS, RS]) extracted the data from the included studies following the completion of full-text screening. Each person extracted their assigned studies and cross-checked the extraction of the other member of the pair. Where available, the following information was extracted from each study: title, author(s), year of publication, study design, length of study, participant demographics (sex, height, weight, age) setting (school/club), level of play, sample size, injury definition, method of injury recording, exposure details (match/training/match and training combined), raw exposure and injury count, injury rate (including 95% confidence intervals [CI]), injury severity, injury burden, injury location, injury type, event causing injury, time in season, time in game, playing position, risk factors examined, analysis method, prevention measures, length of exposure to prevention measure, compliance with intervention, type of analysis undertaken, injury rates in control and intervention groups and effect estimates (including 95% CI). Each study was assessed for quality of evidence (risk of bias) using the Downs and Black quality assessment tool [12]. Each study was scored out of 33 for intervention studies and 25 for non-interventional studies.

### Incidence Rates

Injury incidence rates (IR) as well as concussion incidence rates (CR) were calculated in several different ways, using different injury definitions and rate denominators. For the purposes of this review, rates were calculated for the four most common injury definitions: > 24-h time loss, > 7-day time loss, medical attention and all physical complaints. These were calculated for the three most common denominators: per 1000 h, per 1000 athletic exposures (AEs) and per 1000 participants per season. Rates were produced for matches, training and matches and training combined across four age groups (U12, 12–14 years, 15–18 years, combined) for male and female players and were presented with corresponding 95% CI. U12 (i.e., ≤ 11 years), 12–14 years and 15–18 years were chosen as the three age ranges to represent those in elementary school, junior high and high school. To obtain an accurate pooled estimate of injury rate, the count, raw exposure, injury rate, CI and standard error were required. If one of these values was not reported, it was calculated by the research team using standard methods. If concussion was reported as a proportion of all injuries, but no rate was provided, the rate of concussion was estimated using the overall injury rate, injury count and exposure for the study. If a rate of 0 (i.e., no injuries) was reported for any specific group, this was excluded from the meta-analysis. This was the case for nine match rates, eight training rates and five combined rates for all injuries and once for training concussion rates. Studies that reported just an IR, with no count, exposure, CI, or standard error could not be included in the meta-analysis. This occurred for 18 match rates, 6 training rates, 33 combined rates (largely from one study that did not meet inclusion criteria based on injury definition) and 27 combined concussion rates (largely from the same study). Where standard error and CI were calculated using the raw count and exposure reported in the study (> 100 cases), all calculated rates aligned with what was reported in the study except for two rates from Durie and Munroe [13].

### Descriptive Injury Data

For each of the descriptive characteristics of injury (i.e., severity, burden, location, type, mechanism, time in season, time in game, position), data were presented for the same age groups as rates for males and females. Data were pooled and reported as median and interquartile range (IQR) where sufficient data existed. Where only one study examined a particular finding and no median and IQR were possible, just the proportion was reported. Severity, measured in days lost from sport, was reported in papers as means, medians and in categories (e.g., 1–7 days, 8–28 days, > 28 days) and was thus reported as such. Injury burden was reported when presented in included studies as the product of injury incidence and injury severity. Event associated with injury was described as both specific events (e.g., tackle, ruck, maul) as well as contact and non-contact. For injury type and location, more specific categories were combined into broader categories (e.g., for location: head/neck, upper extremity, trunk, lower extremity).

### Data Synthesis and Analysis

To obtain a pooled estimate for each age group and sex, meta-analyses were conducted using a DerSimonian Laird random effects model [14]. Separate models were used for each subcategory (i.e., U12/12–14 years/15–18 years, male/female, each injury definition, each denominator). In cases where data were presented within a study for individual years, yearly values were included instead of the study mean value. Heterogeneity within pooled estimates was assessed using Q and I^2^ values. Risk factor and prevention studies were reported descriptively and meta-analysis was not conducted due to a small number of studies reporting on a wide range of outcomes. Rates presented in these papers were included in the meta-analysis of rates. All analysis was conducted using STATA 16.0 (Statacorp, College Station, Texas, USA).

## Results

### Identification of Studies

**Fig. 1:**
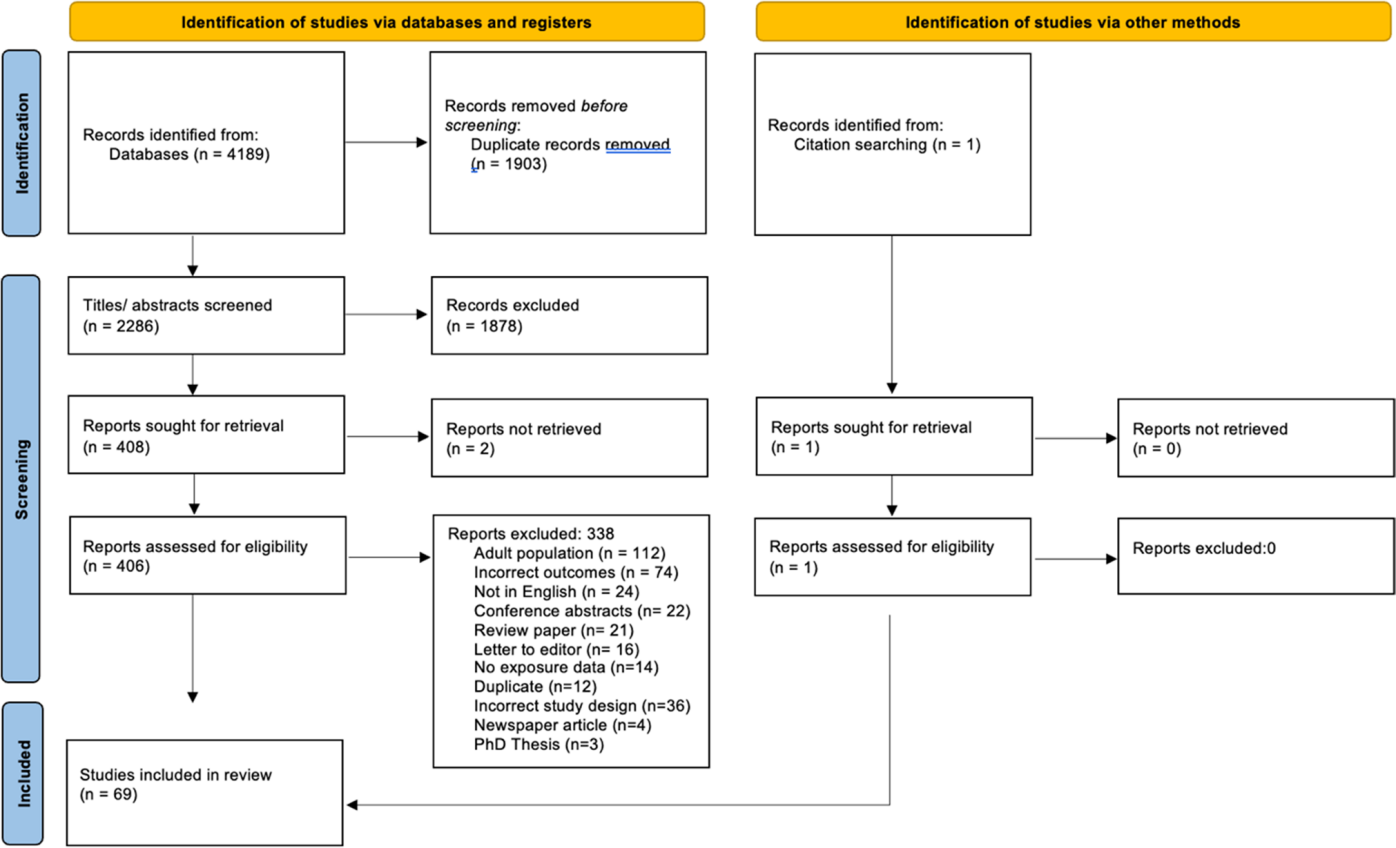
PRISMA flowchart

Initial searches yielded 2286 unique citations (Fig. 1). After initial title and abstract screening, 408 met criteria for full-text review, of which 406 could be retrieved. During full-text review, an additional 338 were excluded, resulting in 69 studies present for the current review (including one found through reference list screening). The top three reasons for exclusion were adult study population (*n* = 112), no injury or concussion outcome (*n* = 74) and not written in English (*n* = 24). The remaining reasons for exclusion are listed in Fig. 1. Inter-rater reliability on the calibration exercise prior to abstract screening between SWW and IJS was 92%. Agreement throughout abstract screening was 92%, while agreement at full-text review was 89%. On review, disagreement between reviewers over inclusion of studies within the full-text screening was largely due to reasons for exclusion, rather than disagreement on the inclusion/exclusion of the study. Details for all included studies (including references of those not cited in text) are presented in Table S1 (see ESM). The mean Downs and Black score for the included studies was 14 (standard deviation [SD] 3). Examination of the Downs and Black score by study design demonstrated the highest mean for randomised controlled trials (*n* = 2; mean 23, SD 1), followed by prospective cohort studies (*n* = 49, mean 13, SD 3), retrospective cohort studies (*n* = 15, mean 13, SD 3), case control studies (*n* = 3, mean 13, SD 2) and ecological studies (*n* = 1, mean 12).

### Injury Incidence Rates

**Fig. 2:**
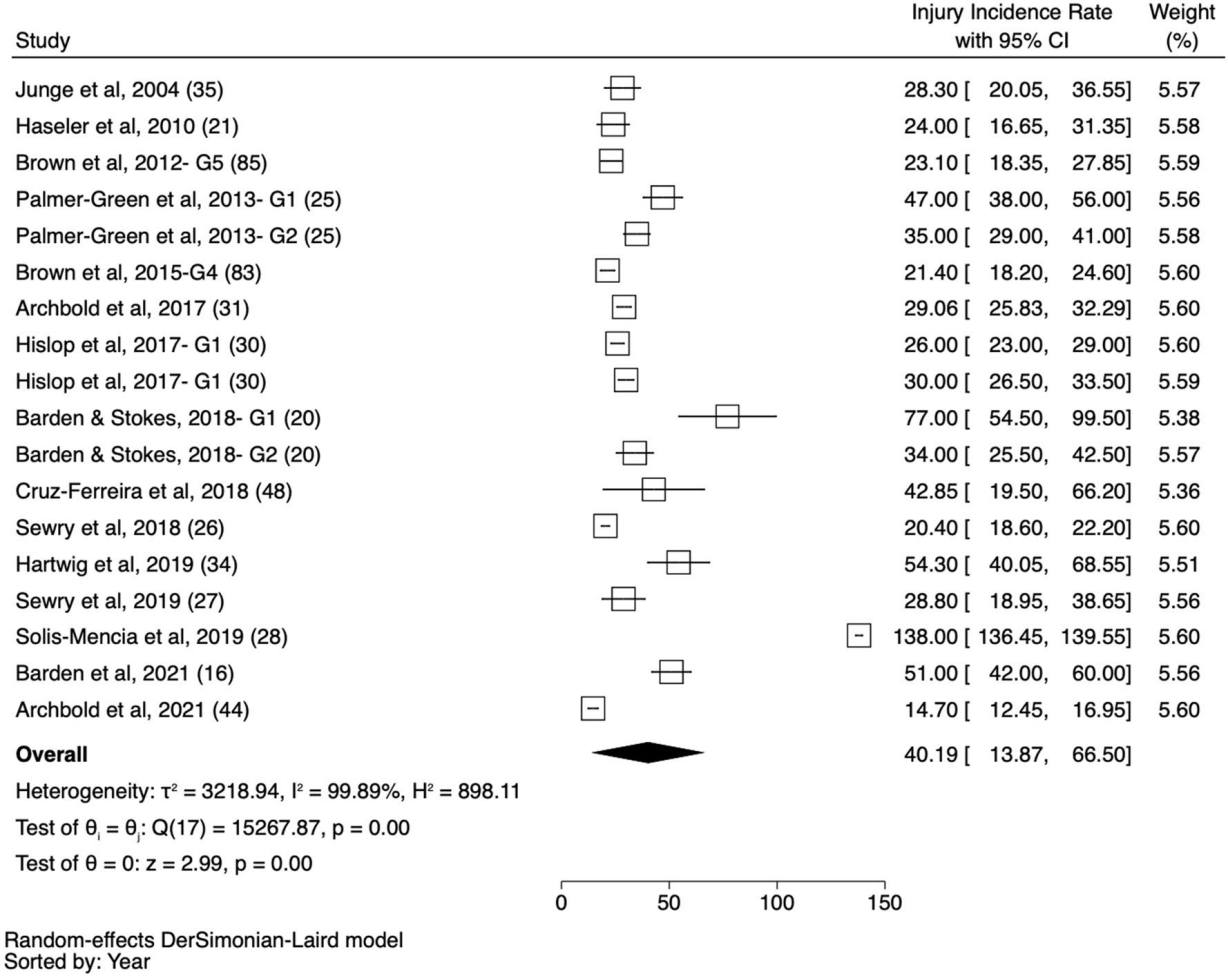
Forest plot of match injury rate* for male players 18 years and under. * Injury rate shown in figure represents the overall injury rate using a 24-h time-loss definition and is reported per 1000 match hours. *G1, G2, G3…*Gx represents each of the unique groups presented in each study

**Fig. 3:**
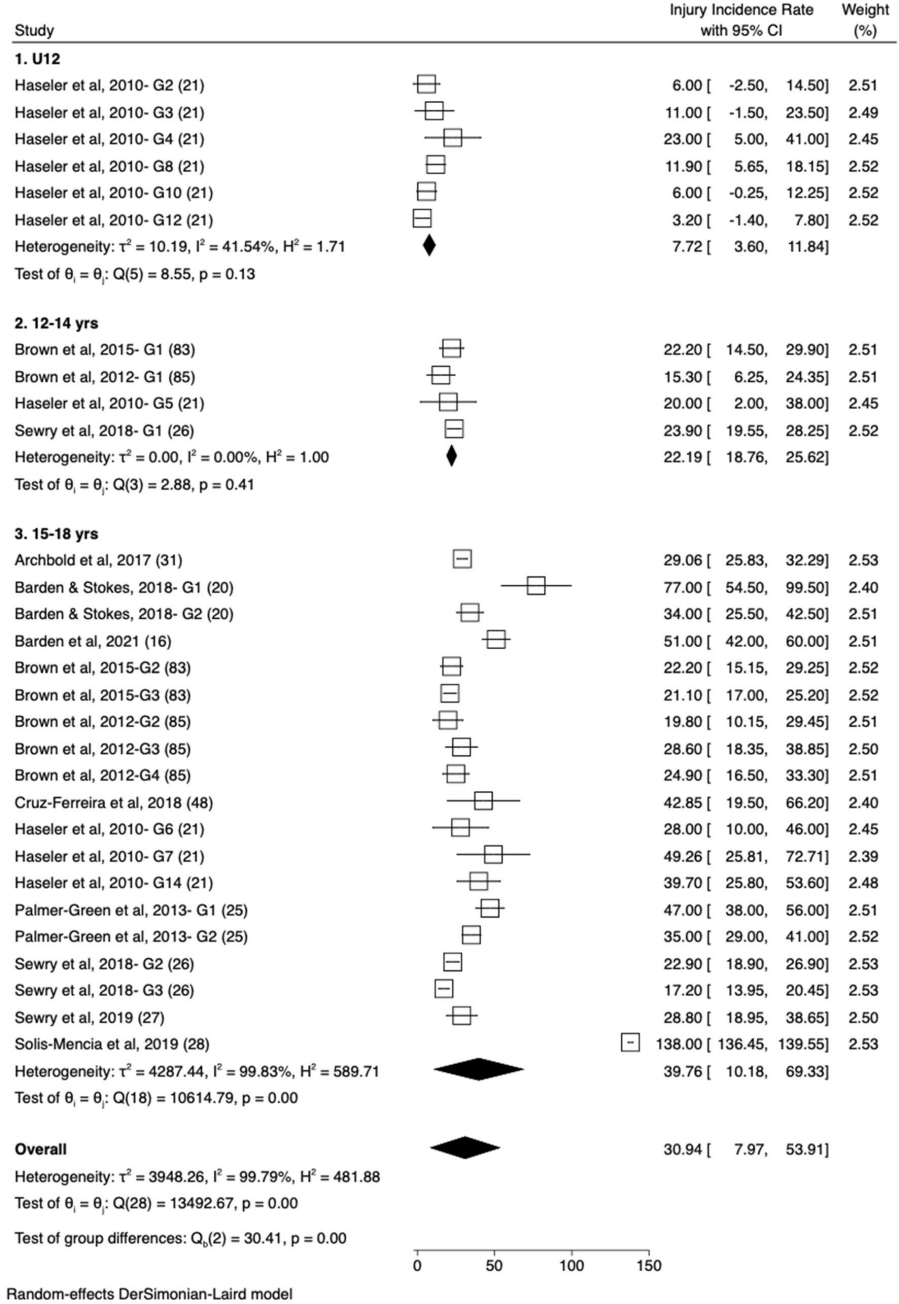
Forest plot of match injury rate* for male players in U12, 12–14 years and 15–18 years categories. * Injury rate shown in figure represents the overall injury rate using a 24-h time-loss definition and is reported per 1000 match hours. *G1, G2, G3*…Gx represents each of the unique groups presented in each study

**Fig. 4:**
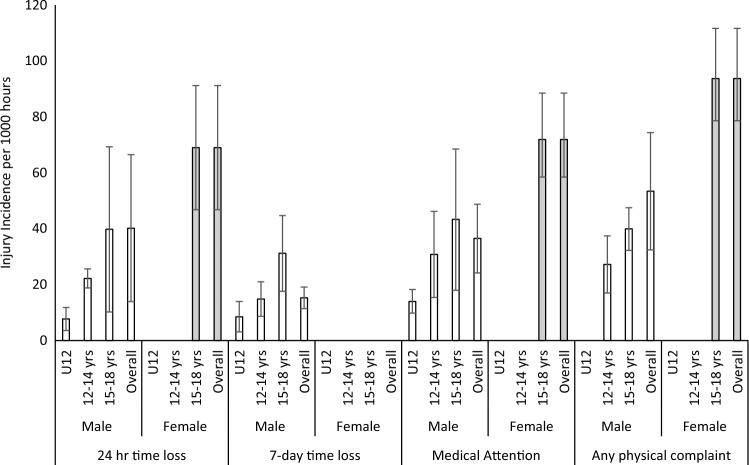
Injury incidence rates using a per 1000-h denominator*. * Rates are presented for males and females across the U12, 12–14 years (years) and 15–18 years age groups for the four most common injury definitions. For exact values, please see Table S2 in the ESM

A total of 285 IRs were extracted from the 69 studies included. There were nine different injury definitions used: 24-h time loss (*n* = 75), all physical complaints (*n* = 53), medical attention (*n* = 52), 7-day time loss (*n* = 46), insurance claims (*n* = 26), any time loss (*n* = 18), medical attention and/or time loss (*n* = 10), catastrophic injuries (*n* = 4) and dental injuries (*n* = 1). Eleven different rate denominators were reported: per 1000 h (*n* = 202), per 1000 players per year (*n* = 50), per 1000 athletic exposures (*n* = 14), per 100,000 players per year (catastrophic injuries, *n* = 6), per 100 player games (*n* = 5), per 100 tackle events (*n* = 2), injuries per hour (*n* = 2), games per injury (*n* = 1), per game (*n* = 1), per school per season (*n* = 1), and per year (*n* = 1). Considering sex, 250 rates included male data only, 24 considered female data, 5 combined male and female and 6 studies did not state the sex of participants. The number of available IRs increased with age: U12 (*n* = 41), 12–14 years (*n* = 63), and 15–18 years (*n* = 108). Pooled data (pooled in the parent study) from these age groups, as well as those that did not fit in one specific age category (*n* = 73) were considered in the overall category (*n* = 147). Forest plots for each definition and denominator were produced, with examples provided in Figs. 2, 3 and 4 for the most common denominator and injury definition in males. To provide comparisons across age, sex and injury definitions, the four most common definitions and three most common denominators were reported in Table S2 (see ESM- See Table S1 for all references of included studies) and Fig. 4. Both the Q statistic and I^2^ statistic indicated heterogeneity in the injury rates reported between included studies (Tables S3 and S4, see ESM).

Irrespective of definition, denominator, sex and age group, match IRs were higher than training IRs. The overall IR in males using a 24-h time-loss definition and a per 1000-h denominator (the most common definition and denominator) were 40.2/1000 h (95% CI 13.9–66.5) for matches and 2.0/1000 h (95% CI 1.5–2.5) for training. For females, the rate was 69.0/1000 h (95% CI 46.8–91.2) for matches and 3.7/1000 h (95% CI 2.6–5.3) for training; however, only two female studies met the inclusion criteria for matches [15, 16] and one for training [15]. In all cases, the 15- to 18-year-old age group demonstrated the highest IR, when compared with U12 and 12–14 years categories. For example, using the same rate definitions and denominators as above, in matches, the rate of injury in boys aged 15–18 years is over fivefold higher than U12 boys (7.7/1000 h [95% CI 3.6–11.8] compared with 39.8/1000 h [95% CI 10.2–69.3]; Table S2 [see ESM], Fig. 4). The higher rate in the 15- to 18-year-old group is consistent for each definition (24-h time loss: 5.2 × greater; 7-day time loss: 3.7 × greater; medical attention: 3.1 × greater; any physical complaint: not possible). To provide a visual representation of the method by which data were pooled, forest plots are provided for the overall IR in U18 males (using the most common definition and denominator: 24-h time loss and per 1000 h; Fig. 2) as well as for the male U12, 12- to 14-year-old group and the 15- to 18-year-old group (Fig. 3). All other IRs were calculated using the same methodology and are presented as just the IRs in Table S2.

To assess the risk of bias within the estimates of IR, two sensitivity checks were undertaken on the most common denominator (per 1000 h) and injury definition (24-h time loss) for males in the ‘overall’ age category. The first included studies that achieved a Downs and Black score of 12 or more only while the second included only studies that were completed after the 2007 consensus statement on injury reporting in rugby [17]. Compared with the IRs inclusive of all relevant studies, there was no evidence of bias in the estimates as neither the sensitivity analysis using the Downs and Black method (score < 11 [34.2/1000 h, 95% CI 19.8–48.6] vs score > 11 [40.2/1000 h, 95% CI 11.9–70.4]) nor the consensus statement method (pre-2007 consensus [28.3/1000 h, 95% CI 20.1–36.6] vs post-2007 consensus [40.9/1000 h, 95% CI 13.7–66.5]) demonstrated a significant difference (based on overlapping CI) in the estimated IR.

Two further exploratory analyses, IRs for the most common denominator (per 1000 h) and injury definition (24-h time loss) for males, were pooled by country. Using these criteria there were five studies from England, four from South Africa, two from Northern Ireland and one each from Portugal, Spain and Australia. The reported IRs were highest in Spain (138/1000 h, 95% CI 136–140), followed by Australia (54/1000 h, 95% CI 40–69), Portugal (43/1000 h, 95% CI 20–66), England (37/1000 h, 95% CI 30–44), Northern Ireland (22/1000 h, 95% CI 8–36) and South Africa (21/1000 h, 95% CI 19–23). Most studies (across all age groups) involved the school setting (*n* = 36), followed by provincial/state (*n* = 9), community club (*n* = 7), all levels (*n* = 6), school and club combined (*n* = 3), academy (*n* = 2) and international (*n* = 2). Using the overall male U18 rate, 24-h time-loss definition and per 1000-h denominator, the reported IRs were highest in international players (91/1000 h, 95% CI 0–184), followed by academy (47/1000 h, 95% CI 38–156), school (35/1000 h, 95% CI 28–42), club (24/1000 h, 95% CI 17–31) and provincial/state (21/1000 h, 95% CI 19–23).

### Concussion Incidence Rates

**Fig. 5:**
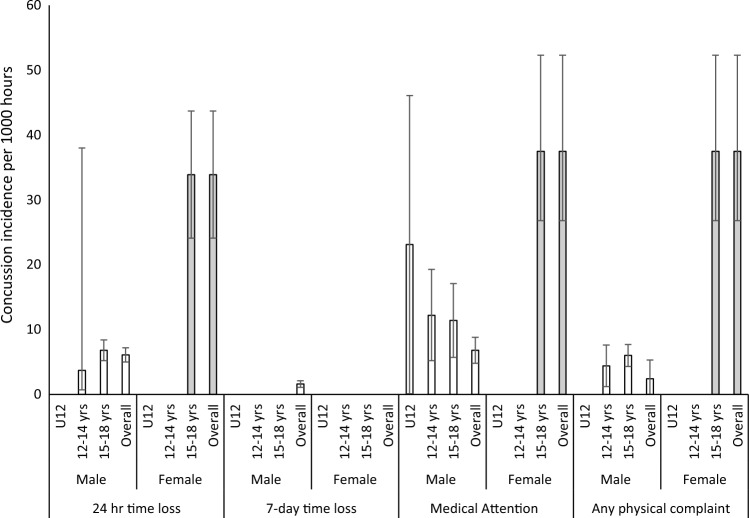
Concussion incidence rates using a per 1000-h denominator*. * Rates are presented for males and females across the U12, 12–14 years (yrs) and 15–18 yrs age groups for the four most common injury definitions. For exact values, please see Table S5 in the ESM

The overall concussion rate (CR) (using the most common 24-h time loss and per 1000 h definitions) was 6.2/1000 h (95% CI 5.0–7.4) for males and 33.9/1000 h (95% CI 24.1–43.7) for females (Table S5 [see ESM], Fig. 5). Compared with overall IRs, the number of available studies reporting CRs was limited, and in several cases, the wide confidence intervals associated with estimates suggest small sample sizes. Given this, comparison between age groups was limited. However, where comparison between age groups was possible, a higher age was associated with a higher injury rate in three out of four injury definitions (Table S5 [see ESM], Fig. 5). Both the *Q* statistic and I^2^ statistic indicated heterogeneity between studies included (Tables S6 and S7, see ESM).

### Descriptive Injury Data

#### Injury Severity

For match injury, the mean time loss for males was 23 days (95% CI 16–29), the median time loss was 21 days (IQR 17–23; Table S8 [see ESM]) and the time-loss category with the highest proportion of injuries was 8–28 days (41%, IQR 36–41) (Table S9 [see ESM]). In females, the mean (40 days), median (17 days) and categorical match injury time loss (highest proportion: 28 + days [47%]) was reported in one study [16].

For training injury, the mean time loss for males was 21 days (95% CI 14–28) and the median was 9 days (Table S8 [see ESM]). No training severity by category was available for males and no training injury severity mean, median, or category was presented for females (Table S9 [see ESM]).

For combined match and training injuries, the mean, median and most common category for males was 63 days, 7 days and 28 + days (62%), respectively (Table S8 [see ESM]). It is important to note that the study that reported mean severity was using a 7-day time-loss injury definition [39]. For females, no mean time loss, median, or categorical severity was presented for combined match and training injuries that met the defined severity categories. More detailed severity values by age group can be seen in Tables S8 and S9 (see ESM).

#### Burden

Match burden of injury was reported in four of the included studies, where four reported burden in males [16, 20, 30, 46] and one reported burden in females [16]. All studies only included participants in the 15–18 age group. Match injury burden was higher in females (2135 days lost/1000 match hours) than males (median: 788.5 days lost/100 match hours, IQR 581–1484; Table S10 [see ESM]). Only one study included the burden of training injury, exclusively in males, and in that study the burden of injury was substantially lower in training than matches (41 days lost/1000 training hours [30]).

#### Injury Location

In matches, the most common injury location was the lower extremity for males (38%, IQR 31–46), whilst the one study in females reported the head/neck as being the most common injury location (50%, IQR 50–51; Table 1). The most common training injury location was the lower extremity in both males (44%, IQR 43–55) and females (39%, IQR 31–38; Table 1). For match and training data combined, lower extremity was once again the location most injured for both males (33%, IQR 26–44) and females (35%, IQR 31–38; Table 1). As highlighted in matches, the proportion of injuries to the head/neck region was 50% (IQR 50–51) in females and 27% (IQR 18–33; Table 1) in males. Injury location by age group can be found in Table S11 (see ESM).

**Table 1:** Injury location for male and female players in match, training and match and training combined

Injury location	Male			Female		
	Match, % (IQR)	Training, % (IQR)	Combined, % (IQR)	Match, % (IQR)	Training, %	Combined, % (IQR)
Head/neck	27 (18–33) [13, 16, 19–31]	9 (5–9) [18, 28]	27 (17–33) [32–44]	50 (50–51) [15, 16]	23 [15]	21 (21–22) [32, 45]
Upper extremity	27 (24–28) [13, 16, 19–31]	15 (14–18) [18, 28]	26 (24–29) [32–44]	18 (16–19) [15, 16]	33 [15]	21 (16–27) [32, 45]
Trunk	8 (7–10) [13, 16, 19–31]	13 (7–23) [18, 28]	8 (7–11) [32–44]	3 (3–3) [15, 16]	5 [15]	2 (1–2) [32, 45]
Lower extremity	38 (31–47) [13, 16, 19–31]	44 (43–55) [18, 28]	33 (26–44) [32–44]	32 (30–33) [15, 16]	39 [15]	35 (31–38) [32, 45]

Data presented represent median proportion of injuries and interquartile range (IQR). Due to the values coming from multiple different studies, the proportion may not sum to 100% total and may be either greater or less than 100%

#### Injury Type

The most common match injury type was ligament injury (33%, IQR 26–37) for males. In females, the central nervous system/peripheral nervous system (CNS/PNS) was the most common (50%), which included concussions; however, this classification was used in just one study [16]. In studies that reported concussion as an isolated grouping, these represented a similar proportion to CNS/PNS (45%, IQR 43–46) (Table S12, see ESM). The most common training injury type was muscle/tendon strain for males (41%, IQR 41–49) (Table S12, see ESM), while in the only female study reporting training data, ligament injuries were the most common type (28% [15]). When match and training injuries were combined, ligament injury was the most common injury type for females (21%) and males (18%, IQR 15–26, Table S12, see ESM). Where comparisons across age groups were possible, in both males and females, the proportion of concussion injuries was higher in older compared with younger age groups (Table S12, see ESM). However, comparisons were not possible across each age group and sex.

#### Mechanism of Injury

Eighty-four percent (IQR 82–87) of male and 89% (IQR 86–92) of female match injuries were associated with a contact event. More specifically, 56% of female and 66% (IQR 63–70) of male injuries occurred during player-to-player contact (Table 2). The tackle event accounted for 55% (IQR 48–57) of all reported male match injuries (tackling: 25%, tackled: 26%; Table 2). For females, 71% of match injuries occurred in the tackle event (41% tackling, 30% tackled [15]). Only one study [18] reported on male training injuries, where 51% occurred during contact and 40% in non-contact mechanisms. In females, this was 75% contact and 21% non-contact (again only reported in one study [15]) More specifically, in males, 28% of all reported injuries occurred during tackle events (tackling: 17%, tackled: 11%; Table 2) and in females 45% of injuries occurred in the tackle (tackling: 24%, tackled: 22%). For match and training combined, 55% (IQR: 52–65) of male injuries occurred in the tackle event (tackling: 27%, tackled: 30%; Table 2). In females where match and training injuries were combined, the tackle event accounted for 62% of injuries (tackling: 33%, tackled: 29%). Injury mechanism by age group can be found in Table S13 (see ESM).

**Table 2:** Event associated with injury for male and female players in match, training and match and training combined

Event associated with injury	Male			Female		
	Match, % (IQR)	Training, %	Combined, % (IQR)	Match, % (IQR)	Training, %	Combined, %
Contact (all)	84 (82–87) [16, 25, 30, 32]	51 [18]	67 [35]	89 (86–92) [15, 16, 32]	75 [15]	N/A
Player contact	66 (63–70) [16, 32]	N/A	N/A	56 [16, 32]	N/A	N/A
Other contact	16 (12–20) [16, 32]	N/A	N/A	28 [16, 32]	N/A	N/A
Non-contact	11 (7–15) [16, 25, 30, 32]	40 [18]	33 [35]	6 (6–7) [15, 16, 32]	21 [15]	N/A
Other	0 [16, 25, 30, 32]	9 [18]	N/A	N/A	4 [15]	N/A
Tackle (all)	55 (48–57) [13, 20–27, 29, 31]	28 [18]	55 (52–65) [28, 32, 36, 37, 39–41, 43, 44]	71 [15]	45 [15]	62 [32]
Tackling	25 (21–29) [13, 20, 22, 23, 25–27, 29, 31]	17 [18]	27 (23–30) [28, 32, 37, 39–41, 43, 44]	41 [15]	24 [15]	33 [32]
Tackled	26 (21–31) [13, 20, 22, 23, 25–27, 29, 31]	11 [18]	30 (25–33) [28, 32, 37, 39–41, 43, 44]	30 [15]	22 [15]	29 [32]
Ruck/maul	14 (12–17) [13, 20–27, 29, 31]	6 [18]	15 (11–18) [28, 32, 36, 37, 39–41, 43, 44]	8 [15]	8 [15]	7 [32]
Other collision	0 (0–0) [13, 20–27, 29, 31]	6 [18]	0 (0–1) [28, 32, 36, 37, 39–41, 43, 44]	5 [15]	9 [15]	0 [32]
Running	3 (0–6) [13, 20–27, 29, 31]	0 [18]	0 (0–0) [28, 32, 36, 37, 39–41, 43, 44]	4 [15]	13 [15]	10 [32]
Scrum	3 (1–7) [13, 20–27, 29, 31]	6 [18]	7 (1–8) [28, 32, 36, 37, 39–41, 43, 44]	5 [15]	5 [15]	0 [32]
Kick	0 (0–0) [13, 20–27, 29, 31]	0 [18]	0 (0–0) [28, 32, 36, 37, 39–41, 43, 44]	0 [15]	0 [15]	0 [32]
Lineout	0 (0–2) [13, 20–27, 29, 31]	0 [18]	1 (0–1) [28, 32, 36, 37, 39–41, 43, 44]	0 [15]	0 [15]	0 [32]
Other	6 (0–11) [13, 20–27, 29, 31]	0 [18]	17 (8–21) [28, 32, 36, 37, 39–41, 43, 44]	4 [15]	15 [15]	0 [32]

Data presented represent median proportion of injuries and interquartile range (IQR). Due to the values coming from multiple different studies, the proportion may not sum to 100% total and may be either greater or less than 100%

#### Time in Season

Four studies evaluated the proportion of injuries that occurred at different times within the season in males [36, 37, 43, 47]. No study reported injury time in season for female participants. Two studies reported match injury [36, 47], one reported training injury [36] and two reported match and training injury combined [36, 37, 43]. For match injuries, 51% were reported to occur in the first half of the season. For training injuries, 70% were reported in the first half of the season. For combined match and training injuries, 60% occurred in the first half of the season.

#### Playing Position

For males, 52% (IQR 50–53 [13, 25, 28, 29, 33, 48]) of match injuries, 36% [28] of training injuries and 49% (IQR 44–55 [28, 36, 37, 40, 41, 43]) of combined match and training injuries were to forwards. No study reported injuries by playing position for females.

#### Time in Game

Eight studies reported injury time in game for males [13, 26–29, 31, 36, 43]. When evaluating the match by quarters, 25% (IQR 20–26), 21% (IQR 17–23), 26% (IQR 24–27) and 26% (IQR 26–28) of injuries occurred in the first, second, third and fourth quarter, respectively. When evaluating the match by halves, 47% (IQR 46–55) and 53% (IQR 46–54) of injuries occurred in the first and second half, respectively [28, 31]. No studies reported injury by time in game for females.

### Risk Factors

**Fig. 6:**
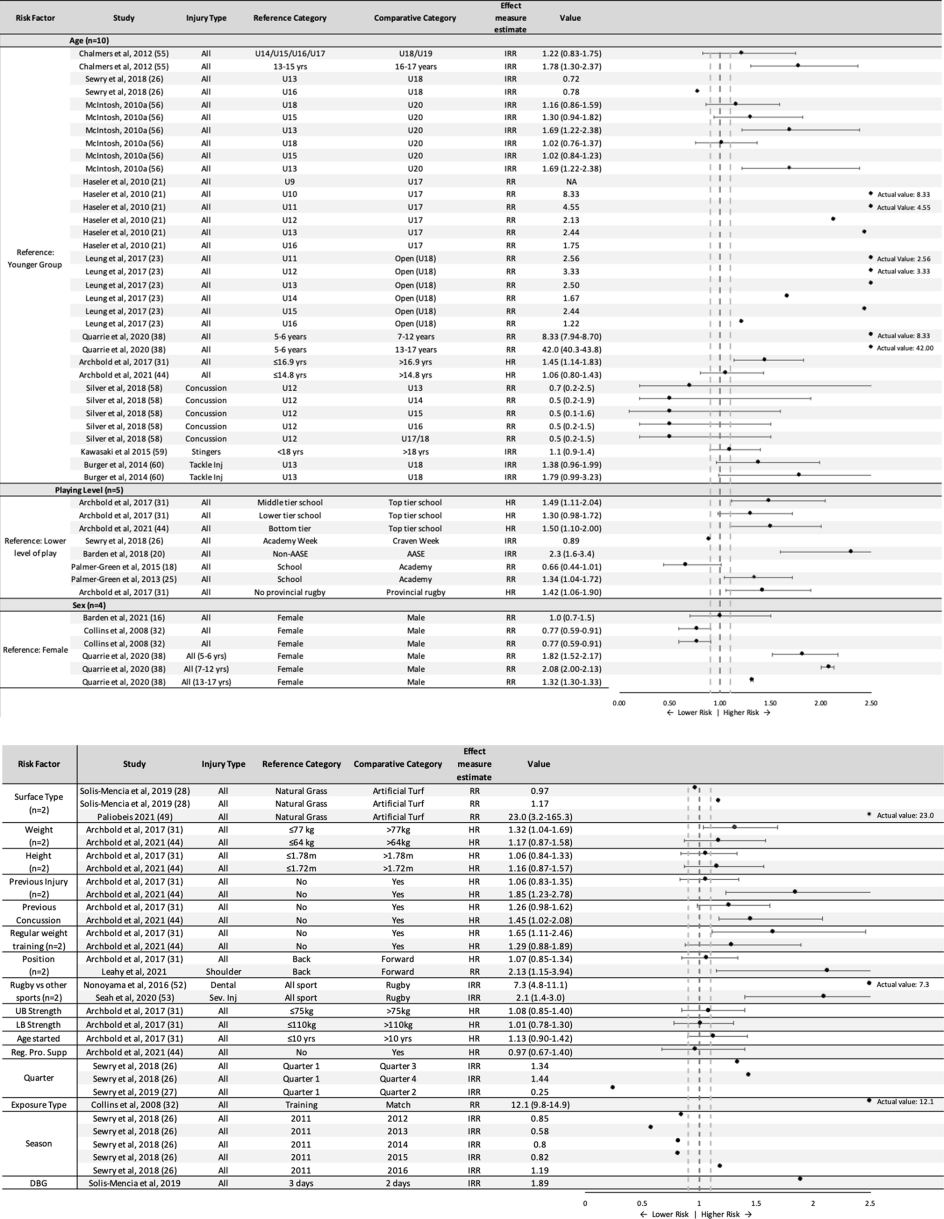
Risk factors by category for all injury and specific injury types reported in the included studies. *AASE* achieving academic and sporting excellence (elite competition [20]), *All* all injuries included, *DBG* days between games, *HR* hazard ratio, *IRR* incidence rate ratio, *LB Strength* lower body strength, *Reg. Pro. Supp* regular protein supplementation, *RR* rate ratio, *Sev. Inj* severe injuries only (injury severity score ≥ 9 [53]), *Tackle inj* tackle injuries only, *UB Strength* upper body strength

Twenty-three injury risk factors were reported in the literature (Fig. 6, Table S15). A further eight risk factors were investigated for tackle-related injuries only (Table S14, see ESM). Risk factors can be considered either modifiable or non-modifiable as well as intrinsic or extrinsic (Table 3). Nineteen risk factors were evaluated using relative risks (RR)/incidence rate ratios (IRR)/hazard ratios (HR) (Fig. 6 and S14, see ESM) and seven were evaluated using odds ratios (OR) (Table S15, see ESM). In 9/11 studies (26/34 comparisons), increasing age was associated with increasing risk of injury (Fig. 6). Similarly, most studies would suggest that a higher level of play is also associated with a greater risk of injury (Fig. 6). Neither males nor females were consistently shown to be at higher risk of injury when examined within the same cohort (Fig. 6). Surface type [28, 49], player weight [31, 44], previous injury [31, 44], previous concussion [31, 44], use of regular weight training [31, 44], position [31, 50], match quarter [26, 27], exposure type [32], season [26], match volume [34], history of shoulder dislocation [51] and rugby itself versus other sports [52, 53] were reported as significant risk factors in at least one study (Fig. 6 and Table S14, see ESM). A number of tackle-related risk factors were also shown to be significant by Burger et al. [54], including match quarter, tackle awareness, initial contact point/first point of contact, ball carrier fend and tackle type (Table S14, see ESM).

**Table 3:** Summary of risk factors for all injuries

	Extrinsic	Intrinsic
Non-modifiable	Position [31, 50, 51] Playing level [18, 20, 22, 25, 26, 31, 44, 51] Exposure type* [32] Season [26] Time in game** [26, 27] Surface type [28, 49]	Sex [16, 32, 38] Age (year or group) [21, 26, 31, 38, 44, 55–61] Height [31, 44] Weight [31, 44] Previous injury history [31, 44] Previous concussion history [31, 44] Previous playing experience [31] Previous shoulder injury [51]
Modifiable	Equipment use*** [31, 44, 62, 63] Days between games [28] Match and training volume [34]	Regular weight training [31, 44] Upper body strength [31] Lower body strength [31] Regular protein supplementation [44]

*Exposure type: match, training.

**Time in game: by quarters.

***Equipment: mouthguard, padded headgear, shoulder pads

### Primary Prevention Strategies

Eight studies evaluating primary prevention strategies were found in this review, with two focusing on law changes [64, 65], four on equipment use [31, 44, 62, 63], one on education [66] and one on neuromuscular training (NMT) [30]. Of these studies, three focused on the prevention of catastrophic injuries [64–66], three on all injury types [30, 62, 63] and four on head/concussion injuries as the primary injury outcome [30, 31, 62, 63]. Both studies examining law changes involved modifications to the scrum sequence [64, 65], and Noakes et al. [64] also examined changes to open-play manoeuvres named the ‘cavalry charge^1^’ and the ‘flying wedge^2^’ in South Africa. The introduction of these policy changes demonstrated conflicting outcomes in each setting, with the changes in South Africa leading to a 46% reduction in spinal cord injuries [64] and the changes in France leading to an increase in spinal cord injuries from 0.4/100,000 players per year to 0.7/100,000 players per year (IRR calculated for the purpose of this review: 1.75 [65]). Of the studies examining protective equipment, no significant difference in injury risk was found with the use of shoulder pads (HR 1.02, 95% CI 0.79–1.30 [31]; HR 1.19, 95% CI 0.89–1.70 [44]). Furthermore, no significant differences were found when using protective headgear for all injury risk (HR 1.07, 95% CI 0.84–1.37 [31]; IRR ranges 0.77–1.09 [63]), head injury risk (IRR ranges 0.84–1.11 [63]; *p* = 0.567 [31]) and concussion specifically (*p* = 0.48 [62]; IRR ranges 0.95–1.13 [63]; *p* = 0.882 [31], HR 1.23, 95% CI 0.89–1.69 [44]). The use of mouthguards was associated with a significant reduction in the risk of injury (HR 0.70, 95% CI 0.54–0.92) and head/face injury specifically (*p* = 0.009 [31]) in U18 players, while in a similar cohort of U15 players, no significant differences in concussion risk were reported (HR 1.69, 95% CI 0.98–2.94) [44]. In the final area of primary prevention, the introduction of a compulsory educational training course for coaches and referees in 2010 was associated with a significant reduction in catastrophic injuries in South Africa (IRR 0.6, 95% CI 0.5–0.8 [66]). Furthermore, the introduction of an NMT programme in high school boys in the UK showed a 72% significant reduction in injury risk (RR 0.28, 90% CI 0.14–0.51) and a 59% significant reduction in concussion (RR 0.41, 90% CI 0.17–0.99) when completed three times per week as recommended [30]. Considering any weekly exposure to this NMT programme, the intervention was associated with reductions in all injury risk (RR 0.85, 95% CI 0.61–1.16) and concussion specifically (RR 0.71, 95% CI 0.48–1.05); however, these were not significant, therefore highlighting the importance of undertaking the programme three time per week as per the programme design. [30].

## Discussion

In this systematic review and meta-analysis, we have aimed to provide a comprehensive guide to the rates, risk factors and primary prevention strategies associated with youth rugby union. To do this, the study considered IRs across both sexes, four age groupings (U12 12–14 years, 15–18 years and overall U18), four injury definitions, three exposure denominators and three exposure types (i.e., match, training, match and training combined). Further to this, injury severity, burden location, type, mechanism and player position have been outlined. Studies reporting risk factors for injury and more specific injury types (e.g., concussion, shoulder injuries, tackle injuries) were included as well as primary prevention strategies across the domains of law/policy change, protective equipment, education and training. This review has highlighted numerous key findings across rates, risk factors and prevention which will be discussed through a lens of identifying common trends, highlighting key gaps and opportunities for future research and prevention efforts.

### Injury and Concussion Rates

There is a substantial body of literature examining IRs in youth rugby broadly, with 69 studies providing 285 unique IRs for this study. Despite this, several limitations exist within the current literature. Firstly, amongst the 285 rates, nine different injury definitions and eleven different denominators have been used. This makes comparison between studies difficult and, as has been previously noted [4], makes it difficult to derive conclusions based on the risk associated with rugby in youth. These methodological differences are evident despite a 2007 consensus statement outlining key collection and reporting considerations for rugby union [17], after which, 65% of the included studies in this review were published. Given these differences, it is unclear whether the range of rates seen may be linked to genuine differences in risk or methodological differences; for example, for male U18 players (24-h time loss and per 1000 h), rates ranged from 15/1000 h [44] to 138/1000 h [28] (a rate ratio between these two populations of 9.4, 95% CI 6.1–14.0). Despite the methodological challenges associated with the included studies, there are two primary findings: (1) the rising risk of injury with age and (2) a lack of epidemiological data related to the female game.

1. Irrespective of the definition or the denominator used, in male youth rugby, the IR rises with each subsequent age grouping (U12 to 12–14 years to 15–18 years). Comparison between age groups in the female game is not possible in the context of these definitions or denominators. This rise in the risk with increasing age is supported by studies examining age as a risk factor, where, in nearly all cases, the older age group was at a greater risk compared with a younger reference group (Fig. 6). This finding is also supported by Quarrie et al. [38], whose insurance claims database demonstrated increasing risk with increasing age (not included in the meta-analysis due to the non-compatibility of injury definitions and denominators with this review) in both males and females. Of particular note, the authors [38] identified the change from 12 to 13 years old (8%) and 13 to 14 years old (9%) as the second and third highest single-year rises in injury risk in females and the change from 12 to 13 years old (7%) and 16 to 17 years old (9%) as the first and second largest single-year rises in risk in males. This age-risk finding likely reflects the changes in the size of players over this period, growing in both height and weight, and in some cases speed, over these years [67]. This increased risk may also be associated with increases in the physicality of the game and, although each country adopts a unique approach to the age of contact introduction, the progression from the touch/tag version of the game to the more contact-intensive game is also around the 10- to 12-year-old age groups.
2. There is a lack of data and evidence surrounding the female game. Using the four primary definitions of injury, only two studies [15, 16] could be included. Outside of these studies, two further studies were found in the review,but were not compatible with definitions outlined in the study [32, 38]. What is notable, however, is that of these four studies examining female data, three were published in either 2020 or 2021, indicating a growing interest in and availability of female data, which offers a promise of further studies in the future. Comparing the rates included in this review, caveated by the inclusion of just two studies, the findings suggest that rates of female injury are higher than those of males. In contrast, when comparing the data presented by Quarrie et al. [38], rates amongst females appear to be consistently lower than those for males across the age categories 5–6 years, 7–12 years and 13–17 years.

Given the increased interest and awareness surrounding concussion in rugby union, and in particular the youth game, pooled CRs were calculated. The pooled CRs calculated for this review (despite a small sample of female papers) indicate a higher risk in females than males; however, when comparing the insurance claim rates by sex presented by Quarrie et al. [38], they indicate similar rates in the 5- to 6-year-old age group, but a higher risk in males at the 7- to 12-year-old and 13- to 17-year-old age groups. The high rates of injury in the studies conducted in female cohorts (both published after 2021) may be due to high levels of public concussion awareness in these populations, and subsequently, improved reporting. However, these differences may also reflect differences in reporting between males and females or genuine differences in injury risk between male and female populations. When comparing concussion rates across age groups, the evidence suggests the rate of concussion increases with each increase in age; however, this occurred in only three of the four possible comparisons included in the meta-analysis as well as in the work of Quarrie et al. [38], and is therefore not conclusive. Interestingly, there was a substantial difference between the CR in male U18 players using the 24-h and 7-day time-loss injury definitions (6.2/1000 h vs 1.6/1000 h). Given the minimum 2-week standdown period recommended by World Rugby before commencing a graduated return to play in youth players [68], the rate of concussion in both the 24-h and 7-day time-loss categories should approximate one another. This finding however, must be interpreted with caution as only two studies used a 7-day time-loss definition and both are > 10 years old [56, 63]. Given the advancements in our understanding of concussion protocols since the publication of these studies, if more recent data were available, these may reflect better alignment between 24-h time-loss and 7-day time-loss concussion rates.

Comparison of concussion or injury rates between sports is challenging given the differences in collection and reporting measures. However, when compared with other youth sports considered to have the highest CRs [9] and using similar definitions and denominators, youth male rugby demonstrated overall injury rates 1.2–3.4 times higher than ice hockey [69], as well as concussion rates 4.7 times higher than ice hockey [70], and 2.8 [71] to 37.0 times higher than American football [72]. Given this, significant primary prevention strategies are required to reduce this risk and ensure the safety and welfare of all youth players across the sport.

### Injury Types and Mechanisms

It is apparent from the available evidence that the tackle is the match event which is associated with most injuries and concussions. When examining the exact mechanisms or characteristics of the tackles associated with concussion, recent work in the adult game has demonstrated differences in specific mechanisms between male (head-to-head) and female (head-to-ground) players [73]. Beyond this, there were no clear findings related to injury aetiology across both sexes, with differences in the most common injury location (lower extremity—males, head/neck—females) and injury type (ligaments—males, CNS/PNS/concussion—females). The apparent differences in the most common type and location of injury between males and females, findings which are limited by the small number of female studies, may demonstrate similar sex-specific differences in head impacts (as reported by Williams et al. [73]); however these comparisons in a youth population are yet to be completed. Injury burden and injury severity (irrespective of reporting method) were higher in females than males. Examining time in game, time in season and playing position, small differences were noted where only single-study evaluation was possible; however, no consistent trends in multiple study comparisons were seen. One notable methodological finding was the widespread variations in the methods used to capture information such as injury location, type and mechanism. This led to the aggregation of certain categories (e.g., injury location) into broad areas (i.e., head/neck, upper extremity, trunk, lower extremity). It is hoped that with the adoption of the recent International Olympic Committee consensus statement for recording and reporting of injury and illness [74] that future studies will be better aligned for comparison between studies. However, what is clear and consistent across sexes and age groups is that the tackle remains a top priority for the game in managing injury and concussion risk.

### Risk Factors for Injury and Concussion

Twenty-three different risk factors for all injury, specific injury types, or specific injury mechanisms were found in this systematic review. Aside from a small number of risk factors, analysis of the association between a risk factor and injury was based on univariable analysis, meaning the effect of confounding or effect modification by other risk factors was not accounted for. Of the risk factors with a larger base of evidence, none showed consistency across studies in their association with injury. However, the weight of evidence did appear to suggest an increase in risk associated with a higher standard of play and the older age groups. Evident within the current research literature is that there is a substantial lack of high-quality multivariable analyses examining injury risk factors in youth rugby. Thus, no firm conclusions or recommendations about potential protective risk factors can be made. Therefore, priority should be placed on targeting high quality risk factor studies in youth rugby union, particularly focused on modifiable risk factors (e.g., physical conditioning, protective equipment, training strategies).

### Prevention of Injury and Concussion

Four primary methods of prevention have been highlighted in the literature: 1) protective equipment 2) policy and law change, 3) training and 4) education. In the context of this systematic review, a total of seven studies were found to investigate prevention strategies in the youth game [30, 31, 62–66], with three focusing exclusively on catastrophic spinal cord injuries [64–66]. Of the four main primary prevention strategies outlined above, only that of training and education demonstrated consistent and significant reductions in injury risk [30, 66]. Of the prevention strategies assessed, the most promising for reducing the risk of concussion specifically was a neuromuscular training (NMT) warm-up programme [30]. However, the evidence for NMT is only apparent in one study. Of note, for both risk factor and prevention strategies, studies have been conducted in male populations only, with the generalisability of findings to the female game currently unknown. Given the high proportion of injuries associated with the tackle, current studies are underway in both France and the United Kingdom to investigate the effect of tackle-related law changes on injury risk in the youth game; however, no studies have currently been published evaluating the outcome of these law variations [75, 76]. Given the high IRs and CRs compared with other youth sports, it is critical that high quality studies focusing on prevention of injuries must be undertaken to reduce injury risk, thereby minimising the deleterious long-term consequences of sports-related injury and concussion whilst maximising the physical and social benefits of team-sport exercise. Given the current evidence, and until further effective interventions become evident, NMT programmes appear to be the most efficacious approach to injury prevention in youth rugby and should be considered as the standard of practice. However, as stated by Hislop et al. [30], efficacy trials in controlled conditions, such as their study, alone are insufficient to assume the real-world effectiveness of this intervention and, as such, further evaluation and adaptation of the programme, particularly in the female game, is required [15, 77]. Importantly though, early studies re-assessing the effectiveness of this intervention appear to be promising, when completed three times per week as prescribed [78].

### Limitations

Several limitations must be considered when interpreting the findings of this systematic review and meta-analyses. A significant limitation of this review was that not all studies were included in the meta-analyses given the heterogeneity in definitions and exposure denominators used to report rates. While every effort to include as many studies as possible was made, the meta-analyses were limited to include four definitions and three denominators only. Furthermore, comparison between males and females and age groups was difficult and must be interpreted with caution, particularly when only a small number of studies could be included. In the case of age categories, many studies reported grouping that could not be included in the categories of U12, 12–14 years and 15–18 years (for example, if a paper reported rates for 13–18 year olds). In these cases, the rates were included in the ‘overall’ (U18) category only. This limited the number of studies that could be included for certain age groups. Finally, several IRs calculated in the meta-analyses indicated significant heterogeneity between studies. IRs were calculated irrespective of evidence of heterogeneity to be thorough and inclusive across age categories, sexes, injury definitions and denominators in this review. The wide range of methods used, countries involved and time periods over which data were collected may have contributed to this heterogeneity between studies. Finally, when evaluating prevention strategies, particularly in relation to concussion, it must be recognised that external influences such as media attention, mandatory stakeholder education and initiatives for improved recognition of concussion (e.g., ‘the Blue Card’ in Australia [79] and Canada [80]) may have impacted injury rates over the evaluation period, masking the benefits of any such prevention strategy [81].

## Conclusions and Recommendations for Future Research

This systematic review has outlined the rates, risk factors and prevention strategies currently available in youth rugby union. Based on the available evidence, there are several key areas requiring attention in the sport. Firstly, the lack of data related to the female game is of particular concern given the rising levels of participation and should be addressed globally. Irrespective of sex, the lack of high quality risk factor studies and injury prevention strategy evaluation indicates that the majority of research in this area to date has focused on establishing the rates and mechanisms of injury (stages 1 and 2 of the van Mechelen sequence of prevention model [10]). Given the IRs and CRs appear to be substantially higher than those of other popular but ‘high-risk’ youth sports, research should prioritise the evaluation and implementation of prevention strategies across protective equipment, laws and policy change, training and education. This must be undertaken as a matter of urgency to minimise the short-term costs of injury and concussion as well as the potential long-term consequences. Furthermore, it is vital that all stakeholders, in particular players and parents, are educated about the risks of playing the sport and appropriate management protocols following injury and concussion specifically. Finally, increased education and awareness of all stakeholders may help in recognising and managing injury and concussion as early as possible to also address secondary and tertiary prevention of injury.

## Supplementary Information

Below is the link to the electronic supplementary material.

Supplementary file1 (DOCX 1349 KB)

## Footnotes

1 “A cavalry charge usually occurs when a penalty kick or free kick is awarded to the attacking team close to the opponent’s goal line. Players of the attacking team line up behind the kicker, spacing themselves across the field in gaps of a metre or two. On receiving a signal from the kicker they begin to charge forward. Only when they are close to the kicker does he tap kick the ball and pass it to one of them. The defending team has to remain behind a line 10 m from the mark or their own goal line (if nearer) until the ball has been kicked.”.

2 “This move usually occurs close to the opponent's goal line when a penalty kick or a free kick is awarded to the attacking team and is initiated by a player either tapping the ball to himself or receiving a short pass, and then driving towards the goal line with his colleagues binding onto either side of him in a V or wedge formation. Frequently the player is isolated illegally by those of his own team in front of him. The dangers inherent in this formation are not for those initiating the move but for those trying to stop it.”.

## Declarations

### Data Availability Statement

There is no dataset publicly available for this study.
