# Supplementary material for: Injury Rates, Mechanisms, Risk Factors and Prevention Strategies in Youth Rugby Union: What’s All the Ruck-Us About? A Systematic Review and Meta-analysis
Source: Sports Med. 2023 May 16;53(7):1375–93. doi: 10.1007/s40279-023-01826-z (PMC10290028; doi:10.1007/s40279-023-01826-z)
Supplement: Supplementary file 1 — Supplementary file1 (DOCX 1349 KB) [file 40279_2023_1826_MOESM1_ESM.docx]

Supplementary Data:

Table S1: Included study details

| **Study** | **Number of participants** | **Age Group** | **Sex** | **Setting** | **Duration** | **Exposure Type** | **Down & Black Score** |
| --- | --- | --- | --- | --- | --- | --- | --- |
| Archbold et al., 2017 [31]: Northern Ireland | 825 | Not reported | Male | School | 1 playing season | Match | 18 |
| Archbold et al., 2018 [19]: Northern Ireland | 825 | 15-18 years | Male | Provincial school’s cup competition | 1 playing season | Match | 12 |
| Archbold et al., 2021 [44]: Northern Ireland | 599 | U15 | Male | School | 1 playing season | Match & training | 15 |
| Barden et al., 2018 [20]: England | 132 | U18, U19 | Male | Academy and school | 3 playing seasons | Match | 14 |
| Barden et al., 2021 [16]: England | Not reported | 16-19 years | Male, Female | School | Male: 4 years  Female: 3 years | Match | 13 |
| Bird et al., 1998 [82]: New Zealand | Male: 54  Female: 23 | Male: U19  Female: U18 | Male, Female | School | 1 playing season | Match & training | 11 |
| Brown et al., 2016 [66]: South Africa | 529,483 each year | Junior | Not reported | Club and academy | 5 playing seasons | Match & training | 12 |
| Brown et al., 2015 [83]; South Africa | 3652 | U13, U16, U18 | Male | Tournament | 2 years | Match | 14 |
| Brown et al., 2013 [84]: South Africa | 121 663 | U7 to U19 | Male, Female | Club and/or school | 4 playing seasons | Match and Training | 15 |
| Brown et al., 2012 [85]: South Africa | 1804 | U13, U16, U18 | Male | Tournament | 1 season of tournaments | Match | 15 |
| Burger et al., 2016 [86]: South Africa | 48 | U18 | Male | Elite rugby union tournament | 3 seasons of week-long tournaments | Match | 13 |
| Burger et al., 2014 [60]: South Africa | 3652 | U13, U16, U18 | Male | Elite rugby union tournament | 2 seasons of week-long tournaments | Match | 13 |
| Burger et al., 2017 [54]: South Africa | 1320 | U18 | Male | Elite rugby union tournament | 3 seasons of week-long tournaments | Match | 17 |
| Carmody et al., 2005 [87]: Australia | Not reported | <18 years | Male | Club, school | 6 years | Match & training | 10 |
| Chalmers et al., 2012 [55]: New Zealand | 704 | 13-18 years | Male | Club | 1 year | Match | 18 |
| Collins et al., 2008 [32]: USA | Male: 98  Female: 23 | 13-19 years | Male, Female | School | 2 playing seasons | Match & training combined | 12 |
| Cruz-Ferreira et al., 2018 [48]: Portugal | 32 | U18 | Male | Club | 1 playing season | Match | 13 |
| Dalley et al., 1980 [88]: New Zealand | 3365 | U14-U19 | Male | Club, school | 1 year | Match | 9 |
| Daniels et al., 2019 [89]: Ireland | 211 | Not reported | Male | School | 1 playing season | Match & Training | 11 |
| Davidson, 1987 [33]: Australia | Not reported | 11-18 years | Male | School | 18 playing seasons | Match | 9 |
| Durie & Munroe, 2000 [13]: New Zealand | 442 | 14-16 years | Male | School | 1 playing season | Match | 10 |
| Garraway et al., 1995 [90]: Scotland | 449 | <16 years, 16-19 years | Male | Club | 1 playing season | Match & training combined | 15 |
| Garraway et al., 2000 [91]: Scotland | 576 player- seasons | <16 years, 16-19 years | Not reported | School | 2 playing season | Match | 16 |
| Hartwig et al., 2019 [34]: Australia | 103 | 14-16 years | Male | School, youth representative | 2 playing seasons | Match & training | 11 |
| Haseler et al., 2010 [21]: England | 210 | U9-U17 | Male | Club | 1 playing season | Match | 8 |
| Hendricks et al., 2016 [92]: South Africa | Not reported | U18 | Male | School | 3 years | Match | 12 |
| Hendricks et al., 2015 [93]: South Africa | Not reported | U18 | Male | School | 3 years | Match | 15 |
| Hislop et al., 2017 [30]: England | 2452 | 14-18 years | Male | School | 1 playing season | Match & training | 24 |
| Junge et al., 2004 [35]: New Zealand | 123 | 14-18 years | Male | School | 1 playing season | Match | 8 |
| Kawasaki et al., 2014 [51]: Japan | 378 | 15-18 years | Male | School, representative teams | 1 playing season | Match & training | 16 |
| Kawasaki et al., 2015 [59]: Japan | 358 | 15-17 years | Male | School, representative teams | 1 playing season | Match | 16 |
| Leahy et al, 2021 [50]: Ireland | 665 | 17-19 years | Male | School | 2 playing seasons | Match | 17 |
| Lee & Garraway, 1996 [22]: Scotland | 1705 | 11-19 years | Male | School, club | 1 playing season | Match | 17 |
| Leung et al., 2017 [23]: Australia | 3585 | 10-18 years | Male | School | 1 playing season | Match | 16 |
| Leung et al., 2017 [57]: Australia | 480 | U11-U19 | Male | School | 1 playing season | Match | 11 |
| Lewis & George, 1996 [47]: England | 45 | U17, U19 | Not reported | Club | 2 playing seasons | Match | 15 |
| Marshall & Spencer, 2001 [94]: USA | Not reported | Not reported | Not reported | School | 3 playing seasons | Match & training | 9 |
| McIntosh & McCrory, 2001 [62]: Australia | 295 | U15 | Male | School | 1 playing season | Match | 13 |
| McIntosh et al., 2009 [63]: Australia | 3868 | U13, U15, U18, U20 | Male | School, club | 2 playing seasons | Match | 22 |
| McIntosh et al., 2010 [56]: Australia | 767 | U13, U15, U18 | Male | School, club | 2 playing seasons | Match | 11 |
| McIntosh et al., 2010 [61]: Australia | Not reported | U15, U18 | Male | School | 5 playing seasons | Match | 16 |
| McManus & Cross, 2004 [36]: Australia | 44 | U15, U16 | Male | State National Championships | 26 weeks | Match & training | 11 |
| Nathan et al., 1983 [37]: South Africa | Not reported | U10, U11, U12, U13, U14, U15, U16, U19 | Male | School | 1 playing season | Match & training | 10 |
| Nicol et al., 2011 [95]: Scotland | 470 | 11-17 years | Male, female | School | 4 months | Match | 13 |
| Noakes et al., 1999 [64]: South Africa | Not reported | Not reported | Male | School | 8 playing seasons | Match & training | 14 |
| Nonoyana et al, 2016 [52]: Japan | 3795 | Not reported | Male | School | 1 year | Match & training combined | 15 |
| Paliobeis et al, 2021 [49]: USA | 3896 total | Not clear | Male, female | School | 2 playing seasons | Match & training combined | 13 |
| Palmer-Green et al., 2015 [18]: England | 472 | 16-18 years | Male | School, academy | 2 years | Training | 18 |
| Palmer-Green et al., 2013 [25]: England | 472 | 16-18 years | Male | School, academy | 2 years | Match | 18 |
| Pringle et al., 1998 [45]: New Zealand | 1932 | 6-15 years | Male | Club | 4 weeks | Match | 12 |
| Quarrie et al., 2020 [38]: New Zealand | Average of 104,289 annually | 5-17 years | Male, female | Club and/or school | 13 years | Match & training combined | 18 |
| Reboursiere et al., 2018 [65]: France | 1,229,468 | 6-18 years | Male, female | Club and/or school | 7 playing seasons | Match & training combined | 19 |
| Roi et al., 2010 [39]: Italy | 350 | 7-14 years | Male | Club | 2 playing seasons | Match & training | 15 |
| Rotem et al., 2001 [96]: Australia | 2320 reported injury | Not clear | Male | School | 30 years | Match | 9 |
| Roux et al., 1987 [40]: South Africa | Not reported | U14, U15, U16, U19 | Male | School | 1 playing season | Match & training | 11 |
| Roux & Noakes, 1993 [41]: South Africa | Not reported | U14, U15, U16, U19 | Male | School | 2 years | Match & training | 11 |
| Seah et al, 2020 [53]: Singapore | 10,951 | 5-17 | Male, female | Club, school | 5 years | Match & training combined | 12 |
| Sewry et al., 2018 [26]: South Africa | Not reported | U13, U16, U19 | Male | Provincial | 4 weeks/year | Match | 17 |
| Sewry et al., 2019 [27]: South Africa | 130 | U16 | Male | School | 1 playing season | Match | 16 |
| Shill et al, 2021 [15]: Canada | 361 | 15-18 | Female | School | 2 playing seasons | Match & training | 16 |
| Shuttleworth-Edwards et al., 2008 [46]: South Africa | 1147 | School | Male | School | Varied (1-2 years) | Match & training combined | 9 |
| Silver et al., 2018 [58]: England | 489 | U9, U10, U11, U12, U13, U14, U15, U16, U17/18 | Not reported | Club | 4 playing seasons | Match | 17 |
| Solis-Mencia et al., 2019 [28]: Spain | 98 | U18 | Male | International | Tournament | Match & training | 18 |
| Sparks, 1981 [42]: England | Not reported | 13-18 years | Male | School | 30 playing seasons | Match & training combined | 10 |
| Sparks, 1985 [43]: England | 2427 | 13-18 years | Male | School | 4 playing seasons | Match & training combined | 10 |
| Sugerman, 1983 [29]: Australia | Not reported | U13, U14, U15, U16, Open | Male | School | 4 months | Match | 12 |
| Takazawa et al., 2016 [97]: Japan | 290 | Not reported | Unclear | School | 11 years | Match & training | 15 |
| Taylor, 2003 [98]: Australia | Not reported | Not reported | Not reported | School | 11 years | Match & training combined | 12 |
| Watson, 1997 [99]: Ireland | 40 | 14-19 years | Male | School level or above | 1 year | Match & training combined | 9 |

Table S2: Pooled injury incidence rates for each definition, denominator, sex, and age group for match, training, and match and training combined.

| **Injury Definition** | **Rate Denominator** | **Exposure Type** | **Male** | | | | **Female** | | | |
| --- | --- | --- | --- | --- | --- | --- | --- | --- | --- | --- |
|  |  |  | **U12** | **12-14** | **15-18** | **Overall** | **U12** | **12-14** | **15-18** | **Overall** |
| **24 hour time loss** | **/ 1000 hours** | **Match (M)** | 7.7  (3.6-11.8)  ^[21]^ | 22.2  (18.8-25.6)  ^[21, 26, 83, 85]^ | 39.8  (10.2-69.3)  ^[16, 20, 21, 25-28, 31, 48, 83, 85]^ | 40.2  (13.9-66.5)  ^[16, 20, 21, 25-28, 30, 31, 34, 35, 44, 48, 83, 85]^ | NA | NA | 69.0  (46.8-91.2)  ^[15, 16]^ | 69.0  (46.8-91.2)  ^[15, 16]^ |
|  |  | **Training (T)** | NA | NA | 1.4  (1.1-1.8)  ^[18, 28]^ | 2.0  (1.5-2.5)  ^[18, 28, 30, 34, 35]^ | NA | NA | 3.7  (2.6-5.3)  ^[15]^ | 3.7  (2.6-5.3)  ^[15]^ |
|  |  | **M & T** | NA | NA | 73.2  (72.5-73.9)  ^[28]^ | 45.9  (0.0-99.5)  ^[28, 34]^ | NA | NA | NA | NA |
|  | **/ 1000 athletic exposures** | **Match (M)** | NA | 105.7  (81.0-130.4) ^[29]^ | 191.9  (103.0-280.9) ^[29]^ | 154.2  (135.1-173.3)  ^[29]^ | NA | NA | NA | NA |
|  |  | **Training (T)** | NA | NA | NA | NA | NA | NA | NA | NA |
|  |  | **M & T** | NA | NA | NA | NA | NA | NA | NA | NA |
|  | **/ 1000 players/ season** | **Match (M)** | NA | NA | NA | NA | NA | NA | NA | NA |
|  |  | **Training (T)** | NA | NA | NA | NA | NA | NA | NA | NA |
|  |  | **M & T** | NA | NA | NA | NA | NA | NA | NA | NA |
| **7-day time loss** | **/ 1000 hours** | **Match (M)** | 8.5  (3.1-14.0)  ^[39]^ | 14.8  (8.7-21.0)  ^[39, 43, 56]^ | 31.2  (17.6-44.7)  ^[43, 56]^ | 15.3  (11.4-19.1)  ^[37, 39-41, 43, 56, 63]^ | NA | NA | NA | NA |
|  |  | **Training (T)** | 0.5  (0.2-0.9)  ^[39]^ | 0.5  (0.1-0.9)  ^[39, 43]^ | 14.5  (11.5-17.4)  ^[43]^ | 0.7  (0.4-1.0)  ^[37, 39-41, 43]^ | NA | NA | NA | NA |
|  |  | **M & T** | 0.8  (0.5-1.0)  ^[37, 39]^ | 2.1  (1.1-3.2)  ^[37, 39, 43]^ | 13.9  (4.2-23.6)  ^[37, 43]^ | 6.3  (5.2-7.4)  ^[37, 39-43]^ | NA | NA | NA | NA |
|  | **/ 1000 athletic exposures** | **Match (M)** | NA | NA | NA | NA | NA | NA | NA | NA |
|  |  | **Training (T)** | NA | NA | NA | NA | NA | NA | NA | NA |
|  |  | **M & T** | NA | NA | NA | NA | NA | NA | NA | NA |
|  | **/ 1000 players/ season** | **Match (M)** | NA | NA | NA | NA | NA | NA | NA | NA |
|  |  | **Training (T)** | NA | NA | NA | NA | NA | NA | NA | NA |
|  |  | **M & T** | NA | NA | NA | NA | NA | NA | NA | NA |
| **Medical attention** | **/ 1000 hours** | **Match (M)** | 14.0  (9.8-18.3)  ^[23, 57]^ | 30.8  (15.4-46.2)  ^[23, 56, 57]^ | 43.3  (18.0-68.5)  ^[23, 56, 57]^ | 36.5  (24.2-48.8)  ^[23, 33, 56, 57, 63, 96]^ | NA | NA | 71.9  (58.5-88.5)  ^[15]^ | 71.9  (58.5-88.5)  ^[15]^ |
|  |  | **Training (T)** | NA | NA | NA | NA | NA | NA | 3.6  (2.8-4.6)  ^[15]^ | 3.6  (2.8-4.6)  ^[15]^ |
|  |  | **M & T** | NA | NA | NA | NA | NA | NA | NA | NA |
|  | **/ 1000 athletic exposures** | **Match (M)** | NA | NA | NA | NA | NA | NA | NA | NA |
|  |  | **Training (T)** | NA | NA | NA | NA | NA | NA | NA | NA |
|  |  | **M & T** | NA | NA | NA | NA | NA | NA | NA | NA |
|  | **/ 1000 players/ season** | **Match (M)** | NA | 7.8  (3.3-12.3)  ^[88]^ | 28.4  (17.2-39.5)  ^[88]^ | 21.2  (11.7-30.7)  ^[88]^ | NA | NA | NA | NA |
|  |  | **Training (T)** | NA | NA | NA | NA | NA | NA | NA | NA |
|  |  | **M & T** | NA | NA | NA | NA | NA | NA | NA | NA |
| **All physical complaints** | **/ 1000 hours** | **Match (M)** | NA | 27.2  (17.0-37.4)  ^[13, 83, 85]^ | 39.9  (32.2-47.5)  ^[13, 83, 85]^ | 53.4  (32.4-74.4)  ^[13, 35, 45, 83, 85]^ | NA | NA | 93.7  (78.6-111.7)  ^[15]^ | 93.7  (78.6-111.7)  ^[15]^ |
|  |  | **Training (T)** | NA | NA | NA | 12.8  (0.0-26.6)  ^[13]^ | NA | NA | 5.3  (4.0-6.9)  ^[15]^ | 5.3  (4.0-6.9)  ^[15]^ |
|  |  | **M & T** | NA | NA | NA | 13.3  (10.3-16.2)  ^[36]^ | NA | NA | NA | NA |
|  | **/ 1000 athletic exposures** | **Match (M)** | NA | NA | NA | NA | NA | NA | NA | NA |
|  |  | **Training (T)** | NA | NA | NA | NA | NA | NA | NA | NA |
|  |  | **M & T** | NA | NA | NA | NA | NA | NA | NA | NA |
|  | **/ 1000 players/ season** | **Match (M)** | NA | NA | NA | NA | NA | NA | NA | NA |
|  |  | **Training (T)** | NA | NA | NA | NA | NA | NA | NA | NA |
|  |  | **M & T** | NA | NA | NA | NA | NA | NA | NA | NA |

Table S3: Pooled concussion incidence rates for each definition, denominator, sex, and age group for match, training, and match and training combined.

| **Injury Definition** | **Rate Denominator** | **Exposure Type** | **Male** | | | | **Female** | | | | |
| --- | --- | --- | --- | --- | --- | --- | --- | --- | --- | --- | --- |
|  |  |  | **U12** | **12-14** | **15-18** | **Overall** | **U12** | **12-14** | **15-18** | **Overall** |  |
| **24 hour time loss** | **/1000 hours** | **Match (M)** | NA | 3.7  (0.7-38.0)  ^[83, 85]^ | 6.8  (5.2-8.4)  ^[16, 20, 31, 83, 85, 92, 93]^ | 6.1  (5.0-7.2)  ^[16, 20, 21, 26-28, 30, 31, 44, 83, 92, 93]^ | NA | NA | 33.9  (24.1-43.7)  ^[15, 16]^ | 33.9  (24.1-43.7)  ^[15, 16]^ |  |
|  |  | **Training (T)** | NA | NA | NA | NA | NA | NA | NA | NA |  |
|  |  | **M & T** | NA | NA | NA | 3.0  (0.6-5.5)  ^[28]^ | NA | NA | NA | NA |  |
|  | **/1000 athletic exposures** | **Match (M)** | NA | NA | NA | 28.7  (20.3-37.2)  ^[29]^ | NA | NA | NA | NA |  |
|  |  | **Training (T)** | NA | NA | NA | NA | NA | NA | NA | NA |  |
|  |  | **M & T** | NA | NA | NA | NA | NA | NA | NA | NA |  |
|  | **/1000 players/ season** | **Match (M)** | NA | NA | NA | NA | NA | NA | NA | NA |  |
|  |  | **Training (T)** | NA | NA | NA | NA | NA | NA | NA | NA |  |
|  |  | **M & T** | NA | NA | NA | NA | NA | NA | NA | NA |  |
| **7-day time loss** | **/1000 hours** | **Match (M)** | NA | NA | NA | 1.6  (1.1-2.1)  ^[56, 63]^ | NA | NA | NA | NA |  |
|  |  | **Training (T)** | NA | NA | NA | NA | NA | NA | NA | NA |  |
|  |  | **M & T** | NA | 2.3  (0.6-4.0)  ^[43]^ | 3.5  (1.3-5.6)  ^[43]^ | 1.2  (0.6-1.9)  ^[37, 39, 42, 43]^ | NA | NA | NA | NA |  |
|  | **/ 1000 athletic exposures** | **Match (M)** | NA | NA | NA | NA | NA | NA | NA | NA |  |
|  |  | **Training (T)** | NA | NA | NA | NA | NA | NA | NA | NA |  |
|  |  | **M & T** | NA | NA | NA | NA | NA | NA | NA | NA |  |
|  | **/ 1000 players/ season** | **Match (M)** | NA | NA | NA | NA | NA | NA | NA | NA |  |
|  |  | **Training (T)** | NA | NA | NA | NA | NA | NA | NA | NA |  |
|  |  | **M & T** | NA | NA | NA | NA | NA | NA | NA | NA |  |
| **Medical attention** | **/1000 hours** | **Match (M)** | 23.1  (0.1-46.1)  ^[58]^ | 12.2  (5.2-19.3)  ^[58]^ | 11.4  (5.7-17.1)  ^[58]^ | 6.8  (4.8-8.8)  ^[23, 56-58, 63]^ | NA | NA | 37.5  (26.8-52.3)  ^[15]^ | 37.5  (36.8-52.8)  ^[15]^ |  |
|  |  | **Training (T)** | NA | NA | NA | NA | NA | NA | NA | NA |  |
|  |  | **M & T** | NA | NA | NA | NA | NA | NA | NA | NA |  |
|  | **/1000 athletic exposures** | **Match (M)** | NA | NA | NA | NA | NA | NA | NA | NA |  |
|  |  | **Training (T)** | NA | NA | NA | NA | NA | NA | NA | NA |  |
|  |  | **M & T** | NA | NA | NA | NA | NA | NA | NA | NA |  |
|  | **/ 000 players/ season** | **Match (M)** | NA | NA | NA | NA | NA | NA | NA | NA |  |
|  |  | **Training (T)** | NA | NA | NA | NA | NA | NA | NA | NA |  |
|  |  | **M & T** | NA | NA | NA | NA | NA | NA | NA | NA |  |
| **All physical complaints** | **/1000 hours** | **Match (M)** | NA | 4.4  (1.2-7.6)  ^[83, 85]^ | 6.0  (4.3-7.7)  ^[83, 85]^ | 2.4  (0.0-5.3)  ^[13, 45, 83]^ | NA | NA | 37.5  (26.8-52.3)  ^[15]^ | 37.5  (36.8-52.8)  ^[15]^ |  |
|  |  | **Training (T)** | NA | NA | NA | NA | NA | NA | NA | NA |  |
|  |  | **M & T** | NA | NA | NA | 1.5  (0.5-2.4)  ^[35]^ | NA | NA | NA | NA |  |
|  | **/ 1000 athletic exposures** | **Match (M)** | NA | NA | NA | NA | NA | NA | NA | NA |  |
|  |  | **Training (T)** | NA | NA | NA | NA | NA | NA | NA | NA |  |
|  |  | **M & T** | NA | NA | NA | NA | NA | NA | NA | NA |  |
|  | **/1000 players/ season** | **Match (M)** | NA | NA | NA | NA | NA | NA | NA | NA |  |
|  |  | **Training (T)** | NA | NA | NA | NA | NA | NA | NA | NA |  |
|  |  | M & T | NA | NA | NA | NA | NA | NA | NA | NA |  |

Table S4: Q-statistic for “All Injury” meta-analysis:

| **Injury Definition** | **Rate Denominator** | **Exposure Type** | **Male** | | | | **Female** | | | | |
| --- | --- | --- | --- | --- | --- | --- | --- | --- | --- | --- | --- |
|  |  |  | **U12** | **12-14** | **15-18** | **Overall** | **U12** | **12-14** | **15-18** | **Overall** |  |
| **24 hour time loss** | **/ 1000 hours** | **Match (M)** | P=0.13 | P=0.41 | p<0.0001 | p<0.0001 | NA | NA | P=0.09 | P=0.09 |  |
|  |  | **Training (T)** | NA | NA | P=0.04 | p<0.0001 | NA | NA | NA | NA |  |
|  |  | **M & T** | NA | NA | NA | p<0.0001 | NA | NA | NA | NA |  |
|  | **/ 1000 athletic exposures** | **Match (M)** | NA | P=0.32 | P=0.003 | NA | NA | NA | NA | NA |  |
|  |  | **Training (T)** | NA | NA | NA | NA | NA | NA | NA | NA |  |
|  |  | **M & T** | NA | NA | NA | NA | NA | NA | NA | NA |  |
|  | **/ 1000 players/ season** | **Match (M)** | NA | NA | NA | NA | NA | NA | NA | NA |  |
|  |  | **Training (T)** | NA | NA | NA | NA | NA | NA | NA | NA |  |
|  |  | **M & T** | NA | NA | NA | NA | NA | NA | NA | NA |  |
| **7-day time loss** | **/ 1000 hours** | **Match (M)** | P=0.84 | p<0.0001 | p<0.0001 | p<0.0001 | NA | NA | NA | NA |  |
|  |  | **Training (T)** | P=0.07 | p<0.0001 | P=0.36 | p<0.0001 | NA | NA | NA | NA |  |
|  |  | **M & T** | P=0.66 | p<0.0001 | p<0.0001 | p<0.0001 | NA | NA | NA | NA |  |
|  | **/ 1000 athletic exposures** | **Match (M)** | NA | NA | NA | NA | NA | NA | NA | NA |  |
|  |  | **Training (T)** | NA | NA | NA | NA | NA | NA | NA | NA |  |
|  |  | **M & T** | NA | NA | NA | NA | NA | NA | NA | NA |  |
|  | **/ 1000 players/ season** | **Match (M)** | NA | NA | NA | NA | NA | NA | NA | NA |  |
|  |  | **Training (T)** | NA | NA | NA | NA | NA | NA | NA | NA |  |
|  |  | **M & T** | NA | NA | NA | NA | NA | NA | NA | NA |  |
| **Medical attention** | **/ 1000 hours** | **Match (M)** | P=0.72 | p<0.0001 | p<0.0001 | p<0.0001 | NA | NA | NA | NA |  |
|  |  | **Training (T)** | NA | NA | NA | NA | NA | NA | NA | NA |  |
|  |  | **M & T** | NA | NA | NA | NA | NA | NA | NA | NA |  |
|  | **/ 1000 athletic exposures** | **Match (M)** | NA | NA | NA | NA | NA | NA | NA | NA |  |
|  |  | **Training (T)** | NA | NA | NA | NA | NA | NA | NA | NA |  |
|  |  | **M & T** | NA | NA | NA | NA | NA | NA | NA | NA |  |
|  | **/ 1000 players/ season** | **Match (M)** | NA | P=0.05 | p<0.0001 | p<0.0001 | NA | NA | NA | NA |  |
|  |  | **Training (T)** | NA | NA | NA | NA | NA | NA | NA | NA |  |
|  |  | **M & T** | NA | NA | NA | NA | NA | NA | NA | NA |  |
| **All physical complaints** | **/ 1000 hours** | **Match (M)** | NA | p<0.0001 | P=0.0009 | p<0.0001 | NA | NA | NA | NA |  |
|  |  | **Training (T)** | NA | NA | NA | p<0.0001 | NA | NA | NA | NA |  |
|  |  | **M & T** | NA | NA | NA | NA | NA | NA | NA | NA |  |
|  | **/ 1000 athletic exposures** | **Match (M)** | NA | NA | NA | NA | NA | NA | NA | NA |  |
|  |  | **Training (T)** | NA | NA | NA | NA | NA | NA | NA | NA |  |
|  |  | **M & T** | NA | NA | NA | NA | NA | NA | NA | NA |  |
|  | **/ 1000 players/ season** | **Match (M)** | NA | NA | NA | NA | NA | NA | NA | NA |  |
|  |  | **Training (T)** | NA | NA | NA | NA | NA | NA | NA | NA |  |
|  |  | **M & T** | NA | NA | NA | NA | NA | NA | NA | NA |  |

Table S5: I^2^ statistic for “All Injury” meta-analysis:

| **Injury Definition** | **Rate Denominator** | **Exposure Type** | **Male** | | | | **Female** | | | | |
| --- | --- | --- | --- | --- | --- | --- | --- | --- | --- | --- | --- |
|  |  |  | **U12** | **12-14** | **15-18** | **Overall** | **U12** | **12-14** | **15-18** | **Overall** |  |
| **24 hour time loss** | **/ 1000 hours** | **Match (M)** | 42% | 0% | 100% | 100% | NA | NA | 65% | 65% |  |
|  |  | **Training (T)** | NA | NA | 70% | 88% | NA | NA | NA | NA |  |
|  |  | **M & T** | NA | NA | NA | 100% | NA | NA | NA | NA |  |
|  | **/ 1000 athletic exposures** | **Match (M)** | NA | 12% | 88% | NA | NA | NA | NA | NA |  |
|  |  | **Training (T)** | NA | NA | NA | NA | NA | NA | NA | NA |  |
|  |  | **M & T** | NA | NA | NA | NA | NA | NA | NA | NA |  |
|  | **/ 1000 players/ season** | **Match (M)** | NA | NA | NA | NA | NA | NA | NA | NA |  |
|  |  | **Training (T)** | NA | NA | NA | NA | NA | NA | NA | NA |  |
|  |  | **M & T** | NA | NA | NA | NA | NA | NA | NA | NA |  |
| **7-day time loss** | **/ 1000 hours** | **Match (M)** | 0% | 92% | 91% | 98% | NA | NA | NA | NA |  |
|  |  | **Training (T)** | 54% | 84% | 0% | 94% | NA | NA | NA | NA |  |
|  |  | **M & T** | 0% | 92% | 97% | 100% | NA | NA | NA | NA |  |
|  | **/ 1000 athletic exposures** | **Match (M)** | NA | NA | NA | NA | NA | NA | NA | NA |  |
|  |  | **Training (T)** | NA | NA | NA | NA | NA | NA | NA | NA |  |
|  |  | **M & T** | NA | NA | NA | NA | NA | NA | NA | NA |  |
|  | **/ 1000 players/ season** | **Match (M)** | NA | NA | NA | NA | NA | NA | NA | NA |  |
|  |  | **Training (T)** | NA | NA | NA | NA | NA | NA | NA | NA |  |
|  |  | **M & T** | NA | NA | NA | NA | NA | NA | NA | NA |  |
| **Medical attention** | **/ 1000 hours** | **Match (M)** | 0% | 97% | 98% | 100% | NA | NA | NA | NA |  |
|  |  | **Training (T)** | NA | NA | NA | NA | NA | NA | NA | NA |  |
|  |  | **M & T** | NA | NA | NA | NA | NA | NA | NA | NA |  |
|  | **/ 1000 athletic exposures** | **Match (M)** | NA | NA | NA | NA | NA | NA | NA | NA |  |
|  |  | **Training (T)** | NA | NA | NA | NA | NA | NA | NA | NA |  |
|  |  | **M & T** | NA | NA | NA | NA | NA | NA | NA | NA |  |
|  | **/ 1000 players/ season** | **Match (M)** | NA | 74% | 91% | 96% | NA | NA | NA | NA |  |
|  |  | **Training (T)** | NA | NA | NA | NA | NA | NA | NA | NA |  |
|  |  | **M & T** | NA | NA | NA | NA | NA | NA | NA | NA |  |
| **All physical complaints** | **/ 1000 hours** | **Match (M)** | NA | 82% | 67% | 98% | NA | NA | NA | NA |  |
|  |  | **Training (T)** | NA | NA | NA | 99% | NA | NA | NA | NA |  |
|  |  | **M & T** | NA | NA | NA | NA | NA | NA | NA | NA |  |
|  | **/ 1000 athletic exposures** | **Match (M)** | NA | NA | NA | NA | NA | NA | NA | NA |  |
|  |  | **Training (T)** | NA | NA | NA | NA | NA | NA | NA | NA |  |
|  |  | **M & T** | NA | NA | NA | NA | NA | NA | NA | NA |  |
|  | **/ 1000 players/ season** | **Match (M)** | NA | NA | NA | NA | NA | NA | NA | NA |  |
|  |  | **Training (T)** | NA | NA | NA | NA | NA | NA | NA | NA |  |
|  |  | M & T | NA | NA | NA | NA | NA | NA | NA | NA |  |

Table S6: Q-statistic for “Concussion only” meta-analysis:

| **Injury Definition** | **Rate Denominator** | **Exposure Type** | **Male** | | | | **Female** | | | | |
| --- | --- | --- | --- | --- | --- | --- | --- | --- | --- | --- | --- |
|  |  |  | **U12** | **12-14** | **15-18** | **Overall** | **U12** | **12-14** | **15-18** | **Overall** |  |
| **24 hour time loss** | **/ 1000 hours** | **Match (M)** | NA | p=0.66 | P=0.003 | p<0.0001 | NA | NA | NA | NA |  |
|  |  | **Training (T)** | NA | NA | NA | NA | NA | NA | NA | NA |  |
|  |  | **M & T** | NA | NA | NA | NA | NA | NA | NA | NA |  |
|  | **/ 1000 athletic exposures** | **Match (M)** | NA | NA | NA | NA | NA | NA | NA | NA |  |
|  |  | **Training (T)** | NA | NA | NA | NA | NA | NA | NA | NA |  |
|  |  | **M & T** | NA | NA | NA | NA | NA | NA | NA | NA |  |
|  | **/ 1000 players/ season** | **Match (M)** | NA | NA | NA | NA | NA | NA | NA | NA |  |
|  |  | **Training (T)** | NA | NA | NA | NA | NA | NA | NA | NA |  |
|  |  | **M & T** | NA | NA | NA | NA | NA | NA | NA | NA |  |
| **7-day time loss** | **/ 1000 hours** | **Match (M)** | NA | NA | NA | P=0.97 | NA | NA | NA | NA |  |
|  |  | **Training (T)** | NA | NA | NA | NA | NA | NA | NA | NA |  |
|  |  | **M & T** | NA | P=0.29 | P=0.09 | p<0.0001 | NA | NA | NA | NA |  |
|  | **/ 1000 athletic exposures** | **Match (M)** | NA | NA | NA | NA | NA | NA | NA | NA |  |
|  |  | **Training (T)** | NA | NA | NA | NA | NA | NA | NA | NA |  |
|  |  | **M & T** | NA | NA | NA | NA | NA | NA | NA | NA |  |
|  | **/ 1000 players/ season** | **Match (M)** | NA | NA | NA | NA | NA | NA | NA | NA |  |
|  |  | **Training (T)** | NA | NA | NA | NA | NA | NA | NA | NA |  |
|  |  | **M & T** | NA | NA | NA | NA | NA | NA | NA | NA |  |
| **Medical attention** | **/ 1000 hours** | **Match (M)** | NA | P=0.92 | P=0.93 | p<0.0001 | NA | NA | NA | NA |  |
|  |  | **Training (T)** | NA | NA | NA | NA | NA | NA | NA | NA |  |
|  |  | **M & T** | NA | NA | NA | NA | NA | NA | NA | NA |  |
|  | **/ 1000 athletic exposures** | **Match (M)** | NA | NA | NA | NA | NA | NA | NA | NA |  |
|  |  | **Training (T)** | NA | NA | NA | NA | NA | NA | NA | NA |  |
|  |  | **M & T** | NA | NA | NA | NA | NA | NA | NA | NA |  |
|  | **/ 1000 players/ season** | **Match (M)** | NA | NA | NA | NA | NA | NA | NA | NA |  |
|  |  | **Training (T)** | NA | NA | NA | NA | NA | NA | NA | NA |  |
|  |  | **M & T** | NA | NA | NA | NA | NA | NA | NA | NA |  |
| **All physical complaints** | **/ 1000 hours** | **Match (M)** | NA | P=0.39 | P=0.56 | p<0.0001 | NA | NA | NA | NA |  |
|  |  | **Training (T)** | NA | NA | NA | NA | NA | NA | NA | NA |  |
|  |  | **M & T** | NA | NA | NA | NA | NA | NA | NA | NA |  |
|  | **/ 1000 athletic exposures** | **Match (M)** | NA | NA | NA | NA | NA | NA | NA | NA |  |
|  |  | **Training (T)** | NA | NA | NA | NA | NA | NA | NA | NA |  |
|  |  | **M & T** | NA | NA | NA | NA | NA | NA | NA | NA |  |
|  | **/ 1000 players/ season** | **Match (M)** | NA | NA | NA | NA | NA | NA | NA | NA |  |
|  |  | **Training (T)** | NA | NA | NA | NA | NA | NA | NA | NA |  |
|  |  | M & T | NA | NA | NA | NA | NA | NA | NA | NA |  |

Table S7: I^2^ statistic for “Concussion only” meta-analysis:

| **Injury Definition** | **Rate Denominator** | **Exposure Type** | **Male** | | | | **Female** | | | | |
| --- | --- | --- | --- | --- | --- | --- | --- | --- | --- | --- | --- |
|  |  |  | **U12** | **12-14** | **15-18** | **Overall** | **U12** | **12-14** | **15-18** | **Overall** |  |
| **24 hour time loss** | **/ 1000 hours** | **Match (M)** | NA | 0% | 60% | 72% | NA | NA | NA | NA |  |
|  |  | **Training (T)** | NA | NA | NA | NA | NA | NA | NA | NA |  |
|  |  | **M & T** | NA | NA | NA | NA | NA | NA | NA | NA |  |
|  | **/ 1000 athletic exposures** | **Match (M)** | NA | NA | NA | NA | NA | NA | NA | NA |  |
|  |  | **Training (T)** | NA | NA | NA | NA | NA | NA | NA | NA |  |
|  |  | **M & T** | NA | NA | NA | NA | NA | NA | NA | NA |  |
|  | **/ 1000 players/ season** | **Match (M)** | NA | NA | NA | NA | NA | NA | NA | NA |  |
|  |  | **Training (T)** | NA | NA | NA | NA | NA | NA | NA | NA |  |
|  |  | **M & T** | NA | NA | NA | NA | NA | NA | NA | NA |  |
| **7-day time loss** | **/ 1000 hours** | **Match (M)** | NA | NA | NA | 0% | NA | NA | NA | NA |  |
|  |  | **Training (T)** | NA | NA | NA | NA | NA | NA | NA | NA |  |
|  |  | **M & T** | NA | 9% | 65% | 98% | NA | NA | NA | NA |  |
|  | **/ 1000 athletic exposures** | **Match (M)** | NA | NA | NA | NA | NA | NA | NA | NA |  |
|  |  | **Training (T)** | NA | NA | NA | NA | NA | NA | NA | NA |  |
|  |  | **M & T** | NA | NA | NA | NA | NA | NA | NA | NA |  |
|  | **/ 1000 players/ season** | **Match (M)** | NA | NA | NA | NA | NA | NA | NA | NA |  |
|  |  | **Training (T)** | NA | NA | NA | NA | NA | NA | NA | NA |  |
|  |  | **M & T** | NA | NA | NA | NA | NA | NA | NA | NA |  |
| **Medical attention** | **/ 1000 hours** | **Match (M)** | NA | 0% | 0% | 85% | NA | NA | NA | NA |  |
|  |  | **Training (T)** | NA | NA | NA | NA | NA | NA | NA | NA |  |
|  |  | **M & T** | NA | NA | NA | NA | NA | NA | NA | NA |  |
|  | **/ 1000 athletic exposures** | **Match (M)** | NA | NA | NA | NA | NA | NA | NA | NA |  |
|  |  | **Training (T)** | NA | NA | NA | NA | NA | NA | NA | NA |  |
|  |  | **M & T** | NA | NA | NA | NA | NA | NA | NA | NA |  |
|  | **/ 1000 players/ season** | **Match (M)** | NA | NA | NA | NA | NA | NA | NA | NA |  |
|  |  | **Training (T)** | NA | NA | NA | NA | NA | NA | NA | NA |  |
|  |  | **M & T** | NA | NA | NA | NA | NA | NA | NA | NA |  |
| **All physical complaints** | **/ 1000 hours** | **Match (M)** | NA | 0% | 0% | 92% | NA | NA | NA | NA |  |
|  |  | **Training (T)** | NA | NA | NA | NA | NA | NA | NA | NA |  |
|  |  | **M & T** | NA | NA | NA | NA | NA | NA | NA | NA |  |
|  | **/ 1000 athletic exposures** | **Match (M)** | NA | NA | NA | NA | NA | NA | NA | NA |  |
|  |  | **Training (T)** | NA | NA | NA | NA | NA | NA | NA | NA |  |
|  |  | **M & T** | NA | NA | NA | NA | NA | NA | NA | NA |  |
|  | **/ 1000 players/ season** | **Match (M)** | NA | NA | NA | NA | NA | NA | NA | NA |  |
|  |  | **Training (T)** | NA | NA | NA | NA | NA | NA | NA | NA |  |
|  |  | M & T | NA | NA | NA | NA | NA | NA | NA | NA |  |

Table S8: Injury Severity (days)- Mean (95% Cis) & Median (IQR)

| Severity reported as | Age Group | Male | | | Female | | |
| --- | --- | --- | --- | --- | --- | --- | --- |
|  |  | Match | Training | Combined | Match | Training | Combined |
| Average | 0-11 yrs | 7 days^[91]^ | N/A | N/A | N/A | N/A | N/A |
|  | 12-14 yrs | 10 days^[91]^ | N/A | N/A | N/A | N/A | N/A |
|  | 15-18 yrs | 24 days (95% CI: 16-32) ^[16, 20, 25, 27, 31, 48, 91]^ | 22 days ^[18]^ | N/A | 40 days ^[16]^ | N/A | N/A |
|  | Overall | 23 days (95% CI: 16-29) ^[16, 20, 22, 25, 27, 30, 31, 48, 91]^ | 21 days (95% CI: 14-28) ^[18, 30]^ | 63 days^[39]^ | 40 days ^[16]^ | N/A | N/A |
| Median | 0-11 yrs | N/A | N/A | N/A | N/A | N/A | N/A |
|  | 12-14 yrs | N/A | N/A | N/A | N/A | N/A | N/A |
|  | 15-18 yrs | 21 days (IQR: 17-23) ^[16, 25, 31]^ | 9 days ^[18]^ | 7 days ^[28]^ | 27 days^[16]^ | N/A | N/A |
|  | Overall | 21 days (IQR: 17-23) ^[16, 25, 31]^ | 9 days ^[18]^ | 7 days ^[28]^ | 27 days^[16]^ | N/A | N/A |

^* Due to the values coming from multiple different studies, the proportion may not sum to 100% total and may be either greater or less than 100%.^

Table S9: Injury Severity (days)- Severity Categories

| Severity reported as | Severity category | Male | | | Female | | | |
| --- | --- | --- | --- | --- | --- | --- | --- | --- |
|  |  | Match | Training | Combined | Match | Training | Combined |  |
| U 11 | | | | | | | | |
| Categories | 1-7 days | N/A | N/A | N/A | N/A | N/A | N/A |  |
|  | 8-21 days | N/A | N/A | N/A | N/A | N/A | N/A |  |
|  | 8-28 days | N/A | N/A | N/A | N/A | N/A | N/A |  |
|  | >28 days | N/A | N/A | N/A | N/A | N/A | N/A |  |
| 12-14 yrs | | | | | | | | |
| Categories | 1-7 days | 76%^[13]^ | N/A | N/A | N/A | N/A | N/A |  |
|  | 8-21 days | 20%^[13]^ | N/A | N/A | N/A | N/A | N/A |  |
|  | 8-28 days | N/A | N/A | N/A | N/A | N/A | N/A |  |
|  | >28 days | 4%^[13]^ | N/A | N/A | N/A | N/A | N/A |  |
| 15-18 yrs | | | | | | | | |
| Categories | 1-7 days | 22% (IQR: 22-46)^[13, 16, 19, 31, 48]^ | N/A | N/A | 3%^[16]^ | N/A | N/A |  |
|  | 8-21 days | 25% ^[13]^ | N/A | N/A | N/A | N/A | N/A |  |
|  | 8-28 days | 39% (IQR: 36-42) ^[16, 19, 31, 48]^ | N/A | N/A | 43%^[16]^ | N/A | N/A |  |
|  | >28 days | 39% (IQR: 36-42) ^[16, 19, 31, 48]^ | N/A | N/A | 47%^[16]^ | N/A | N/A |  |
| Overall | | | | | | | | |
| Categories | 1-7 days | 32% (IQR: 22-45) ^[13, 16, 19, 31, 48, 91]^ | N/A | 47% (IQR: 11-53) ^[35-37, 44, 88]^ | 3%^[16]^ | N/A | N/A |  |
|  | 8-21 days | 23% ^[13]^ | N/A | 21% ^[35, 37]^ | N/A | N/A | N/A |  |
|  | 8-28 days | 41% (IQR: 36-41)^[16, 19, 31, 48, 91]^ | N/A | 62% *^[44]^* | 43%^[16]^ | N/A | N/A |  |
|  | >28 days | 29% (IQR: 14-42) ^[16, 19, 27, 31, 48, 91]^ | N/A | 48% (IQR: 38-59) ^[39, 44]^ | 47%^[16]^ | N/A | N/A |  |

^* Due to the values coming from multiple different studies, the proportion may not sum to 100% total and may be either greater or less than 100%.^

Table S10: Injury Burden

| Age Group | Male | | | Female | | |
| --- | --- | --- | --- | --- | --- | --- |
|  | Match | Training | Combined | Match | Training | Combined |
| 0-11 yrs | N/A | N/A | N/A | N/A | N/A | N/A |
| 12-14 yrs | N/A | N/A | N/A | N/A | N/A | N/A |
| 15-18 yrs | 789 (IQR: 581-1484) ^[16, 20, 30, 46]^ | 41^[30]^ | N/A | 2135^[16]^ | N/A | N/A |
| Overall | 789 (IQR: 581-1484) ^[16, 20, 30, 46]^ | 41^[30]^ | N/A | 2135^[16]^ | N/A | N/A |

^* Due to the values coming from multiple different studies, the proportion may not sum to 100% total and may be either greater or less than 100%.^

Table S11: Injury location by age group

| Injury location | Age group | Male | | | Female | | |
| --- | --- | --- | --- | --- | --- | --- | --- |
|  |  | Match | Training | Combined | Match | Training | Combined |
| Head/ Neck | U12 | N/A | N/A | 5% ^[38]^ | N/A | N/A | 33%^[38]^ |
|  | 12-14 yrs | N/A | N/A | N/A | N/A | N/A | N/A |
|  | 15-18 yrs | 18% (IQR: 16-32)  ^[16, 19, 20, 25, 28]^ | 9% (IQR: 5-9)  ^[18, 28]^ | N/A | 50% (IQR: 50-51)  ^[15, 16]^ | 23% *^[15]^* | N/A |
|  | Overall | 27% (IQR: 18-33)  ^[13, 16, 19-21, 23-31, 91]^ | 9% (IQR: 5-9)  ^[18, 28]^ | 27% (IQR: 17-33)  ^[32-44]^ | 50% (IQR: 50-51)  ^[15, 16]^ | 23% *^[15]^* | 21% (IQR: 21-22)  ^[32, 38]^ |
| Upper Extremity | U12 | N/A | N/A | 59%^[38]^ | N/A | N/A | 26%^[38]^ |
|  | 12-14 yrs | N/A | N/A | N/A | N/A | N/A | N/A |
|  | 15-18 yrs | 24% (IQR: 21-27.5)  ^[16, 19, 20, 25, 28]^ | 15% (IQR: 14-18)  ^[18, 28]^ | N/A | 18% (IQR: 16-19)  ^[15, 16]^ | 33% *^[15]^* | N/A |
|  | Overall | 27% (IQR: 24-28)  ^[13, 16, 19-21, 23-31, 91]^ | 15% (IQR: 14-18)  ^[18, 28]^ | 26% (IQR: 24-29)  ^[32-44]^ | 18% (IQR: 16-19)  ^[15, 16]^ | 33% *^[15]^* | 21% (IQR: 16-27)  ^[32, 38]^ |
| Trunk | U12 | N/A | N/A | 2% ^[38]^ | N/A | N/A | 2%^[38]^ |
|  | 12-14 yrs | N/A | N/A | N/A | N/A | N/A | N/A |
|  | 15-18 yrs | 7% (IQR: 6.5-10)  ^[16, 19, 20, 25, 28]^ | 15% (IQR: 14-18)  ^[18, 28]^ | N/A | 3% (IQR: 3-3)  ^[15, 16]^ | 5% *^[15]^* | N/A |
|  | Overall | 8% (IQR: 7-10)  ^[13, 16, 19-21, 23-31, 91]^ | 13% (IQR: 7-23)  ^[18, 28]^ | 8% (IQR: 7-11)  ^[32-44]^ | 3% (IQR: 3-3)  ^[15, 16]^ | 5% *^[15]^* | 2% (IQR: 1-2)  ^[32, 38]^ |
| Lower Extremity | U12 | N/A | N/A | 32%^[38]^ | N/A | N/A | 47%^[38]^ |
|  | 12-14 yrs | N/A | N/A | N/A | N/A | N/A | N/A |
|  | 15-18 yrs | 47% (IQR: 39.5-50.5)  ^[16, 19, 20, 25, 28]^ | 44% (IQR: 43-55)  ^[18, 28]^ | N/A | 32% (IQR: 30-33)  ^[15, 16]^ | 39% *^[15]^* | N/A |
|  | Overall | 38% (IQR: 31-47)  ^[13, 16, 19-21, 23-31, 91]^ | 44% (IQR: 43-55)  ^[18, 28]^ | 33% (IQR: 26-44)  ^[32-44]^ | 32% (IQR: 30-33)  ^[15, 16]^ | 39% *^[15]^* | 35% (IQR: 31-38)  ^[32, 38]^ |

^* Due to the values coming from multiple different studies, the proportion may not sum to 100% total and may be either greater or less than 100%.^

Table S12: Injury Type

| Injury location | Age group | Male | | | Female | | |
| --- | --- | --- | --- | --- | --- | --- | --- |
|  |  | Match | Training | Combined | Match | Training | Combined |
| Concussion | 0-11 yrs | N/A | N/A | 2%^[38]^ | N/A | N/A | 3% ^[38]^ |
|  | 12-14 yrs | 10% (IQR: 9.5-14.5)  ^[83, 85]^ | N/A | N/A | N/A | N/A | N/A |
|  | 15-18 yrs | 18% (IQR: 12.5-21.75)  ^[19, 27, 28, 31, 83, 85, 92]^ | 0%^[28]^ | 17%  ^[28, 32]^ | 45% (IQR: 43-46)  ^[15, 16]^ | 22% ^[15]^ | 14% ^[32]^ |
|  | Overall | 17% (IQR: 11-24)  ^[13, 16, 19-24, 26-31, 47, 63, 83, 92]^ | 0%^[28]^ | 12% (IQR: 5-38)  ^[28, 32, 34, 37-44]^ | 45% (IQR: 43-46)  ^[15, 16]^ | 22% ^[15]^ | 4% (IQR: 3-9)  ^[32, 38]^ |
| CNS/PNS | 0-11 yrs | N/A | N/A | N/A | N/A | N/A | N/A |
|  | 12-14 yrs | N/A | N/A | N/A | N/A | N/A | N/A |
|  | 15-18 yrs | 12% (IQR: 6.5-25)  ^[16, 20, 25, 27, 31]^ | 7%^[18]^ | N/A | 50%^[16]^ | N/A | N/A |
|  | Overall | 6% (IQR: 4-15)  ^[16, 20, 25-27, 31]^ | 7%^[18]^ | N/A | 50% ^[16]^ | N/A | N/A |
| Bone | 0-11 yrs | N/A | N/A | 17%^[38]^ | N/A | N/A | 18% ^[38]^ |
|  | 12-14 yrs | 8% (IQR: 7-15)  ^[83, 85]^ | N/A | N/A | N/A | N/A | N/A |
|  | 15-18 yrs | 4.5% (IQR: 2.75-6.5)  ^[16, 19, 20, 27, 28, 31, 83, 85]^ | 3% (IQR: 2-10)  ^[18, 28]^ | 16%  ^[28, 32]^ | 10% (IQR: 9-12)  ^[15, 16]^ | 12% ^[15]^ | 10% ^[32]^ |
|  | Overall | 13% (IQR: 6-17)  ^[13, 16, 19, 20, 22-31, 63, 83]^ | 3% (IQR: 2-10)  ^[18, 28]^ | 17% (IQR: 14-26)  ^[28, 32-34, 37-40, 43, 44]^ | 10% (IQR: 9-12)  ^[15, 16]^ | 12% ^[15]^ | 10% (IQR: 10-15)  ^[32, 38]^ |
| Contusion/  Laceration/ Haemotoma | 0-11 yrs | N/A | N/A | 19% ^[38]^ | N/A | N/A | 10% ^[38]^ |
|  | 12-14 yrs | 29% (IQR: 25.5-32)  ^[83, 85]^ | N/A | N/A | N/A | N/A | N/A |
|  | 15-18 yrs | 17.5% (IQR: 13.5-21.25) ^[16, 19, 20, 27, 28, 31, 83, 85]^ | 9% (IQR: 8-12)  ^[18, 28]^ | 11%  ^[28, 32]^ | 8% (IQR: 8-10)  ^[15, 16]^ | 7% ^[15]^ | 9% ^[32]^ |
|  | Overall | 14% (IQR: 9-22)  ^[13, 16, 19, 20, 22-31, 63, 83]^ | 9% (IQR: 8-12)  ^[18, 28]^ | 9% (IQR: 6-15)  ^[28, 32-34, 37-41, 43, 44]^ | 8% (IQR: 8-10)  ^[15, 16]^ | 7% ^[15]^ | 4% (IQR: 4-7)  ^[32, 38]^ |
| Dental | 0-11 yrs | N/A | N/A | 8%^[38]^ | N/A | N/A | 8% ^[38]^ |
|  | 12-14 yrs | 0%  ^[83, 85]^ | N/A | N/A | N/A | N/A | N/A |
|  | 15-18 yrs | 0% | 0%  ^[18, 28]^ | 0%  ^[28, 32]^ | 0%  ^[15, 16]^ | 0% ^[15]^ | 0% ^[32]^ |
|  | Overall | 0 (IQR: 0-0.05)  ^[13, 16, 19, 20, 22-31, 63, 83]^ | 0%  ^[18, 28]^ | 0% (IQR: 0-0%)  ^[28, 32, 34, 37-41, 43, 44]^ | 0%  ^[15, 16]^ | 0% ^[15]^ | 2 (IQR: 1-5)  ^[32, 38]^ |
| Ligament | 0-11 yrs | N/A | N/A | N/A | N/A | N/A | N/A |
|  | 12-14 yrs | 22% (IQR: 20.5-22)  ^[83, 85]^ | N/A | N/A | N/A | N/A | N/A |
|  | 15-18 yrs | 34% (IQR: 32-39.25)  ^[16, 19, 20, 27, 28, 31, 83, 85]^ | 38% (IQR: 35-41)  ^[18, 28]^ | 25%  ^[28, 32]^ | 23% (IQR: 20-24)  ^[15, 16]^ | 28% ^[15]^ | 21% ^[32]^ |
|  | Overall | 32.5% (IQR: 25.75-36.75) ^[13, 16, 19, 20, 22-31, 63, 83]^ | 38% (IQR: 35-41)  ^[18, 28]^ | 18% (IQR: 15-26)  ^[28, 32, 34, 37, 39, 43, 44]^ | 23% (IQR: 20-24)  ^[15, 16]^ | 28% ^[15]^ | 21% ^[32]^ |
| Muscle tendon | 0-11 yrs | N/A | N/A | N/A | N/A | N/A | N/A |
|  | 12-14 yrs | 19% (IQR: 17.5-24)  ^[83, 85]^ | N/A | N/A | N/A | N/A | N/A |
|  | 15-18 yrs | 15% (IQR: 11.5-19.75)  ^[16, 19, 20, 27, 28, 31, 83, 85]^ | 41% (IQR: 41-49)  ^[18, 28]^ | 15%  ^[28, 32]^ | 7% (IQR: 6-10)  ^[15, 16]^ | 15% ^[15]^ | 12% ^[32]^ |
|  | Overall | 12% (IQR: 9-17)  ^[13, 16, 19, 20, 22-31, 63, 83]^ | 41% (IQR: 41-49)  ^[18, 28]^ | 17% (IQR: 13-21)  ^[28, 32, 34, 37, 39-41, 43, 44]^ | 7% (IQR: 6-10)  ^[15, 16]^ | 15% ^[15]^ | 12% ^[32]^ |
| Visceral | 0-11 yrs | N/A | N/A | 0%^[38]^ | N/A | N/A | 0%^[38]^ |
|  | 12-14 yrs | 0%  ^[83, 85]^ | N/A | N/A | N/A | N/A | N/A |
|  | 15-18 yrs | 0%  ^[16, 19, 20, 27, 28, 31, 83, 85]^ | 0%^[18, 28]^ | 0%  ^[28, 32]^ | 0%  ^[15, 16]^ | 0% ^[15]^ | 0% ^[32]^ |
|  | Overall | 0%  ^[13, 16, 19, 20, 22-31, 63, 83]^ | 0%^[18, 28]^ | 0% (IQR: 0-0)  ^[28, 32, 34, 37-41, 43, 44]^ | 0%  ^[15, 16]^ | 0% ^[15]^ | 0% ^[32]^ |
| Other | 0-11 yrs | N/A | N/A | 0%^[38]^ | N/A | N/A | 4% ^[38]^ |
|  | 12-14 yrs | 0% (IQR: 0-4)  ^[83, 85]^ | N/A | N/A | N/A | N/A | N/A |
|  | 15-18 yrs | 0%  ^[16, 19, 20, 27, 28, 31, 83, 85]^ | 3% (IQR: 2-6)  ^[18, 28]^ | 0%  ^[28, 32]^ | 9% (IQR: 5-10)  ^[15, 16]^ | 15% ^[15]^ | 0% ^[32]^ |
|  | Overall | 2% (IQR: 0-5)  ^[13, 16, 19, 20, 22-31, 63, 83]^ | 3% (IQR: 2-6)  ^[18, 28]^ | 1% (IQR: 0-3)  ^[28, 32, 34, 37-41, 43, 44]^ | 9% (IQR: 5-10)  ^[15, 16]^ | 15% ^[15]^ | 2% (IQR: 1-2)  ^[32, 38]^ |

^* Due to the values coming from multiple different studies, the proportion may not sum to 100% total and may be either greater or less than 100%.^

Table S13: Mechanism of Injury

| Injury location | Age group | Male | | | Female | | |
| --- | --- | --- | --- | --- | --- | --- | --- |
|  |  | Match | Training | Combined | Match | Training | Combined |
| Contact (all) | 0-11 yrs | N/A | N/A | N/A | N/A | N/A | N/A |
|  | 12-14 yrs | N/A | N/A | N/A | N/A | N/A | N/A |
|  | 15-18 yrs | 81% ^[16]^ | 51%^[18]^ | N/A | 91% (IQR: 89-93)  ^[15, 16]^ | 75% *^[15]^* | N/A |
|  | Overall | 84% (IQR: 82-87)  ^[16, 25, 30, 32]^ | 51%^[18]^ | 67%^[35]^ | 89% (IQR: 86-92)  ^[15, 16, 32]^ | 75% *^[15]^* | N/A |
| Player contact | 0-11 yrs | N/A | N/A | N/A | N/A | N/A | N/A |
|  | 12-14 yrs | N/A | N/A | N/A | N/A | N/A | N/A |
|  | 15-18 yrs | 73%^[16]^ | N/A | N/A | 60% ^[16]^ | N/A | N/A |
|  | Overall | 66% (IQR: 63-70)  ^[16, 32]^ | N/A | N/A | 56%  ^[16, 32]^ | N/A | N/A |
| Other contact | 0-11 yrs | N/A | N/A | N/A | N/A | N/A | N/A |
|  | 12-14 yrs | N/A | N/A | N/A | N/A | N/A | N/A |
|  | 15-18 yrs | 8%^[16]^ | N/A | N/A | 27% ^[16]^ | N/A | N/A |
|  | Overall | 16% (IQR: 12-20)  ^[16, 32]^ | N/A | N/A | 28%  ^[16, 32]^ | N/A | N/A |
| Non-contact | 0-11 yrs | N/A | N/A | N/A | N/A | N/A | N/A |
|  | 12-14 yrs | N/A | N/A | N/A | N/A | N/A | N/A |
|  | 15-18 yrs | 16%^[16]^ | 40%^[18]^ | N/A | 6% (IQR: 6-8)  ^[15, 16]^ | 21% *^[15]^* | N/A |
|  | Overall | 11% (IQR: 7-15)  ^[16, 25, 30, 32]^ | 40%^[18]^ | 33%^[35]^ | 6% (IQR: 6-7)  ^[15, 16, 32]^ | 21% *^[15]^* | N/A |
| Other | 0-11 yrs | N/A | N/A | N/A | N/A | N/A | N/A |
|  | 12-14 yrs | N/A | N/A | N/A | N/A | N/A | N/A |
|  | 15-18 yrs | 3%^[16]^ | 9%^[18]^ | N/A | 3%^[16]^ | N/A | N/A |
|  | Overall | 0%  ^[16, 25, 30, 32]^ | 9%^[18]^ | N/A | N/A | N/A | N/A |
|  | 0-11 yrs | N/A | N/A | N/A | N/A | N/A | N/A |
|  | 12-14 yrs | N/A | N/A | N/A | N/A | N/A | N/A |
| Tackle (all) | 15-18 yrs | 55% (IQR: 51-56)  ^[20, 25, 31]^ | 28%^[18]^ | 73%^[28]^ | 71% ^[15]^ | 45% ^[15]^ | N/A |
|  | Overall | 55% (IQR: 48-57)  ^[13, 20-27, 29, 31]^ | 28%^[18]^ | 55% (IQR: 52-65)  ^[28, 32, 36, 37, 39-41, 43, 44]^ | 71% ^[15]^ | 45% ^[15]^ | 62%^[32]^ |
| Tackling | 0-11 yrs | N/A | N/A | N/A | N/A | N/A | N/A |
|  | 12-14 yrs | N/A | N/A | N/A | N/A | N/A | N/A |
|  | 15-18 yrs | 25% (IQR: 21-28)  ^[20, 25, 31]^ | 17%^[18]^ | 28%^[28]^ | 41% ^[15]^ | 24% ^[15]^ | N/A |
|  | Overall | 25% (IQR: 21-29)  ^[13, 20, 22, 23, 25-27, 29, 31]^ | 17%^[18]^ | 27% (IQR: 23-30)  ^[28, 32, 37, 39-41, 43, 44]^ | 41% ^[15]^ | 24% ^[15]^ | 33%^[32]^ |
| Tackled | 0-11 yrs | N/A | N/A | N/A | N/A | N/A | N/A |
|  | 12-14 yrs | N/A | N/A | N/A | N/A | N/A | N/A |
|  | 15-18 yrs | 30% (IQR: 26-32)  ^[20, 25, 31]^ | 11%^[18]^ | 45%^[28]^ | 30% ^[15]^ | 22% ^[15]^ | N/A |
|  | Overall | 26% (IQR: 21-31)  ^[13, 20, 22, 23, 25-27, 29, 31]^ | 11%^[18]^ | 30% (IQR: 25-33)  ^[28, 32, 37, 39-41, 43, 44]^ | 30% ^[15]^ | 22% ^[15]^ | 29%^[32]^ |
| Ruck/maul | 0-11 yrs | N/A | N/A | N/A | N/A | N/A | N/A |
|  | 12-14 yrs | N/A | N/A | N/A | N/A | N/A | N/A |
|  | 15-18 yrs | 14% (IQR: 12-16)  ^[20, 25, 31]^ | 6%^[18]^ | 13%^[28]^ | 8% ^[15]^ | 8% ^[15]^ | N/A |
|  | Overall | 14% (IQR: 12-17)  ^[13, 20-27, 29, 31]^ | 6%^[18]^ | 15% (IQR: 11-18)  ^[28, 32, 36, 37, 39-41, 43, 44]^ | 8% ^[15]^ | 8% ^[15]^ | 7%^[32]^ |
| Other collision | 0-11 yrs | N/A | N/A | N/A | N/A | N/A | N/A |
|  | 12-14 yrs | N/A | N/A | N/A | N/A | N/A | N/A |
|  | 15-18 yrs | 7% (IQR: 6-9)  ^[20, 25, 31]^ | 6%^[18]^ | 3%^[28]^ | 5% ^[15]^ | 9% ^[15]^ | N/A |
|  | Overall | 0% (IQR: 0-0)  ^[13, 20-27, 29, 31]^ | 6%^[18]^ | 0% (IQR: 0-1)  ^[28, 32, 36, 37, 39-41, 43]^ | 5% ^[15]^ | 9% ^[15]^ | 0%^[32]^ |
| Running | 0-11 yrs | N/A | N/A | N/A | N/A | N/A | N/A |
|  | 12-14 yrs | N/A | N/A | N/A | N/A | N/A | N/A |
|  | 15-18 yrs | 0% (IQR: 0-3)  ^[20, 25, 31]^ | 0%^[18]^ | 0%^[28]^ | 4% ^[15]^ | 13% ^[15]^ | N/A |
|  | Overall | 3% (IQR: 0-6)  ^[13, 20-27, 29, 31]^ | 0%^[18]^ | 0% (IQR: 0-0)  ^[28, 32, 36, 37, 39-41, 43]^ | 4% ^[15]^ | 13% ^[15]^ | 10%^[32]^ |
| Scrum | 0-11 yrs | N/A | N/A | N/A | N/A | N/A | N/A |
|  | 12-14 yrs | N/A | N/A | N/A | N/A | N/A | N/A |
|  | 15-18 yrs | 3% (IQR: 2-3)  ^[20, 25, 31]^ | 6%^[18]^ | 7%^[28]^ | 5% ^[15]^ | 5% ^[15]^ | N/A |
|  | Overall | 3% (IQR: 1-7)  ^[13, 20-27, 29, 31]^ | 6%^[18]^ | 7% (IQR: 1-8)  ^[28, 32, 36, 37, 39-41, 43, 44]^ | 5% ^[15]^ | 5% ^[15]^ | 0%^[32]^ |
| Kick | 0-11 yrs | N/A | N/A | N/A | N/A | N/A | N/A |
|  | 12-14 yrs | N/A | N/A | N/A | N/A | N/A | N/A |
|  | 15-18 yrs | 0% (IQR: 0-0)  ^[20, 25, 31]^ | 0%^[18]^ | 0%^[28]^ | 0% ^[15]^ | 0% ^[15]^ | N/A |
|  | Overall | 0% (IQR: 0-0)  ^[13, 20-27, 29, 31]^ | 0%^[18]^ | 0% (IQR: 0-0)  ^[28, 32, 36, 37, 39-41, 43]^ | 0% ^[15]^ | 0% ^[15]^ | 0%^[32]^ |
| Lineout | 0-11 yrs | N/A | N/A | N/A | N/A | N/A | N/A |
|  | 12-14 yrs | N/A | N/A | N/A | N/A | N/A | N/A |
|  | 15-18 yrs | 0% (IQR: 0-0)  ^[20, 25, 31]^ | 0%^[18]^ | 0%^[28]^ | 0% ^[15]^ | 0% ^[15]^ | N/A |
|  | Overall | 0% (IQR: 0-2)  ^[13, 20-27, 29, 31]^ | 0%^[18]^ | 1% (IQR: 0-1)  ^[28, 32, 36, 37, 39-41, 43, 44]^ | 0% ^[15]^ | 0% ^[15]^ | 0%^[32]^ |
| Other | 0-11 yrs | N/A | N/A | N/A | N/A | N/A | N/A |
|  | 12-14 yrs | N/A | N/A | N/A | N/A | N/A | N/A |
|  | 15-18 yrs | 0% (IQR: 0-12)  ^[20, 25, 31]^ | 0% ^[18]^ | 0%^[28]^ | 4% ^[15]^ | 15% ^[15]^ | N/A |
|  | Overall | 6% (IQR: 0-11)  ^[13, 20-27, 29, 31]^ | 0%^[18]^ | 17% (IQR: 8-21)  ^[28, 32, 36, 37, 39-41, 43]^ | 4% ^[15]^ | 15% ^[15]^ | 0%^[32]^ |

^* Due to the values coming from multiple different studies, the proportion may not sum to 100% total and may be either greater or less than 100%.^

Table S14: Risk Factors for tackle related injuries

^^

^**Abbreviations: Tackle Inj: Tackle injuries only; IC: Initial contact; FPOC: First Point of Contact; RR: Rate ratio^

Table S15: Risk Factors for injury (studies reporting Odds Ratios)


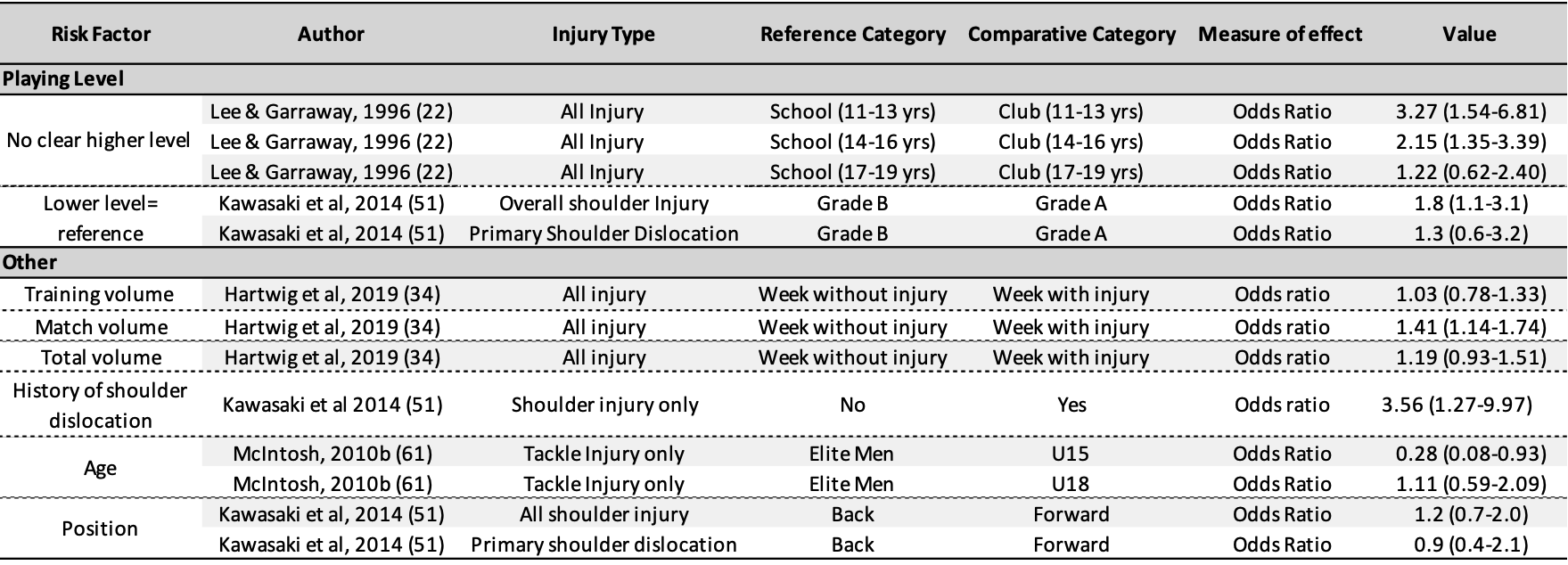


**Appendix 1:**

**Rates, risk factors and prevention strategies for injury in school-based youth rugby: A systematic review and meta-analysis**

FINAL SEARCHES

ORIGINAL searches conducted September 2020

UPDATE searches conducted January 2022

**MEDLINE(R) and Epub Ahead of Print, In-Process, In-Data-Review & Other Non-Indexed Citations and Daily (OVID)** 1946 to December 30, 2021 

| **#** | **Searches** |
| --- | --- |
| 1 | rugby.mp. |
| 2 | Adolescent/ |
| 3 | Young Adult/ |
| 4 | exp Child/ |
| 5 | Schools/ |
| 6 | (child* or adolescen* or youth* or teen* or juvenile* or junior or minor*).tw,kf. |
| 7 | (schoolboy* or school-boy* or schoolgirl* or school-girl*).tw,kf. |
| 8 | (boy* or girl*).tw,kf. |
| 9 | (U9 or U10 or U11 or U12 or U13 or U14 or U15 or U16 or U17 or U18 or U19 or U20 or U21).tw,kf. |
| 10 | (under adj2 ("9" or "10" or "11" or "12" or "13" or "14" or "15" or "16" or "17" or "18" or "19" or "20" or "21")).tw,kf. |
| 11 | (age* adj2 ("9*" or "10*" or "11*" or "12*" or "13*" or "14*" or "15*" or "16*" or "17*" or "18*")).tw,kf. |
| 12 | (("9*" or "10*" or "11*" or "12*" or "13*" or "14*" or "15*" or "16*" or "17*" or "18*") adj2 (year* or yr*)).tw,kf. |
| 13 | (young adj2 (player* or athlete* or adult*)).tw,kf. |
| 14 | (youth adj2 (academy or player* or athlete*)).tw,kf. |
| 15 | (pediatric* or paediatric*).tw,kf. |
| 16 | school*.tw,kf. |
| 17 | or/2-14 |
| 18 | Athletic Injuries/ |
| 19 | exp "Sprains and Strains"/ or Rupture/ or Rotator Cuff Injuries/ |
| 20 | brain injuries, traumatic/ or brain concussion/ or head injuries, closed/ |
| 21 | craniocerebral trauma/ or brain injuries/ |
| 22 | exp Fractures, Bone/ |
| 23 | exp Facial Injuries/ or exp Neck Injuries/ or exp Shoulder Injuries/ or exp Tooth Injuries/ |
| 24 | arm injuries/ or back injuries/ or leg injuries/ or ankle injuries/ or femoral fractures/ or foot injuries/ or tibial fractures/ |
| 25 | Peripheral Nerve Injuries/ or Soft Tissue Injuries/ |
| 26 | knee injuries/ or anterior cruciate ligament injuries/ or knee dislocation/ or patellar dislocation/ or tibial meniscus injuries/ |
| 27 | Lacerations/ or Contusions/ or Hematoma/ |
| 28 | in.fs. |
| 29 | injur*.tw,kf. |
| 30 | (concuss* or mtbi*).tw,kf. |
| 31 | (head adj2 (trauma or contact or impact*)).tw,kf. |
| 32 | (sprain* or strain* or ruptur*).tw,kf. |
| 33 | (fracture* or break or broke* or dislocat* or dis-locat*).tw,kf. |
| 34 | (laceration* or contusion* or bruis* or hematoma* or haematoma*).tw,kf. |
| 35 | or/18-33 |
| 36 | 1 and 17 and 35 |

**Embase (OVID)** 1974 to 2021 December 30 

| **#** | **Searches** |
| --- | --- |
| 1 | rugby/ |
| 2 | rugby.tw,kw. |
| 3 | or/1-2 |
| 4 | adolescent/ |
| 5 | young adult/ |
| 6 | child/ or boy/ or girl/ |
| 7 | exp school child/ or high school student/ or high school/ or exp middle school student/ or exp middle school/ or school/ |
| 8 | (child* or adolescen* or youth* or teen* or juvenile* or junior or minor*).tw,kw. |
| 9 | (schoolboy* or school-boy* or schoolgirl* or school-girl*).tw,kw. |
| 10 | (boy* or girl*).tw,kw. |
| 11 | (U9 or U10 or U11 or U12 or U13 or U14 or U15 or U16 or U17 or U18 or U19 or U20 or U21).tw,kw. |
| 12 | (under adj2 ("9" or "10" or "11" or "12" or "13" or "14" or "15" or "16" or "17" or "18" or "19" or "20" or "21")).tw,kw. |
| 13 | (age* adj2 ("9*" or "10*" or "11*" or "12*" or "13*" or "14*" or "15*" or "16*" or "17*" or "18*")).tw,kw. |
| 14 | (("9*" or "10*" or "11*" or "12*" or "13*" or "14*" or "15*" or "16*" or "17*" or "18*") adj2 (year* or yr*)).tw,kw. |
| 15 | (young adj2 (player* or athlete* or adult*)).tw,kw. |
| 16 | (youth adj2 (academy or player* or athlete*)).tw,kw. |
| 17 | (pediatric* or paediatric*).tw,kw. |
| 18 | school*.tw,kw. |
| 19 | or/4-18 |
| 20 | sport injury/ |
| 21 | sprain/ or musculoskeletal injury/ or muscle strain/ or rupture/ or ligament rupture/ or tendon rupture/ or exp rotator cuff injury/ |
| 22 | traumatic brain injury/ or pediatric traumatic brain injury/ |
| 23 | brain concussion/ or brain injury/ or concussion/ or head injury/ |
| 24 | exp fracture/ |
| 25 | exp face injury/ or exp neck injury/ or exp shoulder injury/ or exp tooth injury/ or exp arm injury/ |
| 26 | leg injury/ or ankle injury/ or foot injury/ or hip injury/ or knee injury/ or leg fracture/ |
| 27 | peripheral nerve injury/ or soft tissue injury/ |
| 28 | anterior cruciate ligament injury/ or knee ligament injury/ or anterior cruciate ligament rupture/ |
| 29 | knee dislocation/ or patella dislocation/ or knee meniscus rupture/ |
| 30 | laceration/ or contusion/ or hematoma/ |
| 31 | injur*.tw,kw. |
| 32 | (concuss* or mtbi*).tw,kw. |
| 33 | (head adj2 (trauma or contact or impact*)).tw,kw. |
| 34 | (sprain* or strain* or ruptur*).tw,kw. |
| 35 | (fracture* or break or broke* or dislocat* or dis-locat*).tw,kw. |
| 36 | (laceration* or contusion* or bruis* or hematoma* or haematoma*).tw,kw. |
| 37 | or/20-35 |
| 38 | 3 and 19 and 37 |
| 39 | limit 38 to conference abstracts |
| 40 | 38 not 39 |

**APA PsycInfo (OVID)** 1806 to December Week 4 2021 

| **#** | **Searches** |
| --- | --- |
| 1 | rugby.mp. |
| 2 | schools/ or elementary schools/ or high schools/ or junior high schools/ or middle schools/ |
| 3 | (child* or adolescen* or youth* or teen* or juvenile* or junior or minor*).tw,id. |
| 4 | (schoolboy* or school-boy* or schoolgirl* or school-girl*).tw,id. |
| 5 | (boy* or girl*).tw,id. |
| 6 | (U9 or U10 or U11 or U12 or U13 or U14 or U15 or U16 or U17 or U18 or U19 or U20 or U21).tw,id. |
| 7 | (under adj2 ("9" or "10" or "11" or "12" or "13" or "14" or "15" or "16" or "17" or "18" or "19" or "20" or "21")).tw,id. |
| 8 | (age* adj2 ("9*" or "10*" or "11*" or "12*" or "13*" or "14*" or "15*" or "16*" or "17*" or "18*")).tw,id. |
| 9 | (("9*" or "10*" or "11*" or "12*" or "13*" or "14*" or "15*" or "16*" or "17*" or "18*") adj2 (year* or yr*)).tw,id. |
| 10 | (young adj2 (player* or athlete* or adult*)).tw,id. |
| 11 | (youth adj2 (academy or player* or athlete*)).tw,id. |
| 12 | (pediatric* or paediatric*).tw,id. |
| 13 | school*.tw,id. |
| 14 | or/2-13 |
| 15 | injuries/ |
| 16 | brain injuries/ or traumatic brain injury/ or head injuries/ or brain concussion/ |
| 17 | injur*.tw,id. |
| 18 | (concuss* or mtbi*).tw,id. |
| 19 | (head adj2 (trauma or contact or impact*)).tw,id. |
| 20 | (sprain* or strain* or ruptur*).tw,id. |
| 21 | (fracture* or break or broke* or dislocat* or dis-locat*).tw,id. |
| 22 | (laceration* or contusion* or bruis* or hematoma* or haematoma*).tw,id. |
| 23 | or/15-22 |
| 24 | 1 and 14 and 23 |

**EBM Reviews - Cochrane Database of Systematic Reviews (OVID)** 2005 to December 28, 2021 

| **#** | **Searches** |
| --- | --- |
| 1 | rugby.tw,kw. |
| 2 | (child* or adolescen* or youth* or teen* or juvenile* or junior or minor*).tw,kw. |
| 3 | (schoolboy* or school-boy* or schoolgirl* or school-girl*).tw,kw. |
| 4 | (boy* or girl*).tw,kw. |
| 5 | (U9 or U10 or U11 or U12 or U13 or U14 or U15 or U16 or U17 or U18 or U19 or U20 or U21).tw,kw. |
| 6 | (under adj2 ("9" or "10" or "11" or "12" or "13" or "14" or "15" or "16" or "17" or "18" or "19" or "20" or "21")).tw,kw. |
| 7 | (age* adj2 ("9*" or "10*" or "11*" or "12*" or "13*" or "14*" or "15*" or "16*" or "17*" or "18*")).tw,kw. |
| 8 | (("9*" or "10*" or "11*" or "12*" or "13*" or "14*" or "15*" or "16*" or "17*" or "18*") adj2 (year* or yr*)).tw,kw. |
| 9 | (young adj2 (player* or athlete* or adult*)).tw,kw. |
| 10 | (youth adj2 (academy or player* or athlete*)).tw,kw. |
| 11 | (pediatric* or paediatric*).tw,kw. |
| 12 | school*.tw,kw. |
| 13 | or/2-12 |
| 14 | injur*.tw,kw. |
| 15 | (concuss* or mtbi*).tw,kw. |
| 16 | (head adj2 (trauma or contact or impact*)).tw,kw. |
| 17 | (sprain* or strain* or ruptur*).tw,kw. |
| 18 | (fracture* or break or broke* or dislocat* or dis-locat*).tw,kw. |
| 19 | (laceration* or contusion* or bruis* or hematoma* or haematoma*).tw,kw. |
| 20 | or/14-19 |
| 21 | 1 and 13 and 20 |

**EBM Reviews - Cochrane Central Register of Controlled Trials (OVID)** November 2021 

| **#** | **Searches** |
| --- | --- |
| 1 | rugby.mp. |
| 2 | Adolescent/ |
| 3 | Young Adult/ |
| 4 | exp Child/ |
| 5 | Schools/ |
| 6 | (child* or adolescen* or youth* or teen* or juvenile* or junior or minor*).tw,kw. |
| 7 | (schoolboy* or school-boy* or schoolgirl* or school-girl*).tw,kw. |
| 8 | (boy* or girl*).tw,kw. |
| 9 | (U9 or U10 or U11 or U12 or U13 or U14 or U15 or U16 or U17 or U18 or U19 or U20 or U21).tw,kw. |
| 10 | (under adj2 ("9" or "10" or "11" or "12" or "13" or "14" or "15" or "16" or "17" or "18" or "19" or "20" or "21")).tw,kw. |
| 11 | (age* adj2 ("9*" or "10*" or "11*" or "12*" or "13*" or "14*" or "15*" or "16*" or "17*" or "18*")).tw,kw. |
| 12 | (("9*" or "10*" or "11*" or "12*" or "13*" or "14*" or "15*" or "16*" or "17*" or "18*") adj2 (year* or yr*)).tw,kw. |
| 13 | (young adj2 (player* or athlete* or adult*)).tw,kw. |
| 14 | (youth adj2 (academy or player* or athlete*)).tw,kw. |
| 15 | (pediatric* or paediatric*).tw,kw. |
| 16 | school*.tw,kw. |
| 17 | or/2-14 |
| 18 | Athletic Injuries/ |
| 19 | exp "Sprains and Strains"/ or Rupture/ or Rotator Cuff Injuries/ |
| 20 | brain injuries, traumatic/ or brain concussion/ or head injuries, closed/ |
| 21 | craniocerebral trauma/ or brain injuries/ |
| 22 | exp Fractures, Bone/ |
| 23 | exp Facial Injuries/ or exp Neck Injuries/ or exp Shoulder Injuries/ or exp Tooth Injuries/ |
| 24 | arm injuries/ or back injuries/ or leg injuries/ or ankle injuries/ or femoral fractures/ or foot injuries/ or tibial fractures/ |
| 25 | Peripheral Nerve Injuries/ or Soft Tissue Injuries/ |
| 26 | knee injuries/ or anterior cruciate ligament injuries/ or knee dislocation/ or patellar dislocation/ or tibial meniscus injuries/ |
| 27 | Lacerations/ or Contusions/ or Hematoma/ |
| 28 | in.fs. |
| 29 | injur*.tw,kw. |
| 30 | (concuss* or mtbi*).tw,kw. |
| 31 | (head adj2 (trauma or contact or impact*)).tw,kw. |
| 32 | (sprain* or strain* or ruptur*).tw,kw. |
| 33 | (fracture* or break or broke* or dislocat* or dis-locat*).tw,kw. |
| 34 | (laceration* or contusion* or bruis* or hematoma* or haematoma*).tw,kw. |
| 35 | or/18-33 |
| 36 | 1 and 17 and 35 |

**CINAHL PLUS with Full Text (Ebsco)**

| **#** | **Query** | **Limiters/Expanders** |
| --- | --- | --- |
| S1 | (MH "Rugby") | Search modes - Find all my search terms |
| S2 | rugby | Search modes - Find all my search terms |
| S3 | S1 OR S2 | Search modes - Find all my search terms |
| S4 | (MH "Adolescence") OR (MH "Child") OR (MH "Young Adult") | Search modes - Find all my search terms |
| S5 | (MH "Schools, Elementary") OR (MH "Schools, Middle") OR (MH "Schools, Secondary") OR (MH "Schools") | Search modes - Find all my search terms |
| S6 | TI ( (child* or adolescen* or youth* or teen* or juvenile* or junior or minor*) ) OR AB ( (child* or adolescen* or youth* or teen* or juvenile* or junior or minor*) ) | Search modes - Find all my search terms |
| S7 | TI ( (schoolboy* or school-boy* or schoolgirl* or school-girl*) ) OR AB ( (schoolboy* or school-boy* or schoolgirl* or school-girl*) ) | Search modes - Find all my search terms |
| S8 | TI ( (boy* or girl*) ) OR AB ( (boy* or girl*) ) | Search modes - Find all my search terms |
| S9 | TI ( (U9 or U10 or U11 or U12 or U13 or U14 or U15 or U16 or U17 or U18 or U19 or U20 or U21) ) OR AB ( (U9 or U10 or U11 or U12 or U13 or U14 or U15 or U16 or U17 or U18 or U19 or U20 or U21) ) | Search modes - Find all my search terms |
| S10 | TI ( (under N2 ("9" or "10" or "11" or "12" or "13" or "14" or "15" or "16" or "17" or "18" or "19" or "20" or "21")) ) OR AB ( (under N2 ("9" or "10" or "11" or "12" or "13" or "14" or "15" or "16" or "17" or "18" or "19" or "20" or "21")) ) | Search modes - Find all my search terms |
| S11 | TI ( (age* N2 ("9*" or "10*" or "11*" or "12*" or "13*" or "14*" or "15*" or "16*" or "17*" or "18*")) ) OR AB ( (age* N2 ("9*" or "10*" or "11*" or "12*" or "13*" or "14*" or "15*" or "16*" or "17*" or "18*")) ) | Search modes - Find all my search terms |
| S12 | TI ( (("9*" or "10*" or "11*" or "12*" or "13*" or "14*" or "15*" or "16*" or "17*" or "18*") N2 (year* or yr*)) ) OR AB ( (("9*" or "10*" or "11*" or "12*" or "13*" or "14*" or "15*" or "16*" or "17*" or "18*") N2 (year* or yr*)) ) | Search modes - Find all my search terms |
| S13 | TI ( (young N2 (player* or athlete* or adult*)) ) OR AB ( (young N2 (player* or athlete* or adult*)) ) | Search modes - Find all my search terms |
| S14 | TI ( (youth N2 (academy or player* or athlete*)) ) OR AB ( (youth N2 (academy or player* or athlete*)) ) | Search modes - Find all my search terms |
| S15 | TI ( (pediatric* or paediatric*) ) OR AB ( (pediatric* or paediatric*) ) | Search modes - Find all my search terms |
| S16 | TI school* OR AB school* | Search modes - Find all my search terms |
| S17 | S4 OR S5 OR S6 OR S7 OR S8 OR S9 OR S10 OR S11 OR S12 OR S13 OR S14 OR S15 OR S16 | Search modes - Find all my search terms |
| S18 | (MH "Athletic Injuries") OR (MH "Rugby Injuries") | Search modes - Find all my search terms |
| S19 | (MH "Sprains and Strains+") OR (MH "Rupture+") OR (MH "Rotator Cuff Injuries") | Search modes - Find all my search terms |
| S20 | (MH "Brain Concussion") OR (MH "Head Injuries") OR (MH "Brain Injuries") | Search modes - Find all my search terms |
| S21 | (MH "Fractures+") | Search modes - Find all my search terms |
| S22 | (MH "Facial Injuries") OR (MH "Tooth Injuries") OR (MH "Neck Injuries") | Search modes - Find all my search terms |
| S23 | (MH "Arm Injuries+") OR (MH "Back Injuries+") OR (MH "Leg Injuries+") | Search modes - Find all my search terms |
| S24 | (MH "Soft Tissue Injuries") | Search modes - Find all my search terms |
| S25 | (MH "Contusions and Abrasions") OR (MH "Dislocations+") OR (MH "Ligament Injuries+") OR (MH "Tears and Lacerations+") | Search modes - Find all my search terms |
| S26 | (MH "Knee Injuries") OR (MH "Knee Dislocation") OR (MH "Anterior Cruciate Ligament Injuries") OR (MH "Knee Injuries, Articular Cartilage") OR (MH "Medial Collateral Ligament Sprain") OR (MH "Meniscal Injuries") OR (MH "Posterior Cruciate Ligament Injuries") | Search modes - Find all my search terms |
| S27 | (MH "Femoral Fractures+") OR (MH "Knee Fractures+") OR (MH "Tibial Fractures+") | Search modes - Find all my search terms |
| S28 | TI injur* OR AB injur* | Search modes - Find all my search terms |
| S29 | TI ( (concuss* or mtbi*) ) OR AB ( (concuss* or mtbi*) ) | Search modes - Find all my search terms |
| S30 | TI ( (head N2 (trauma or contact or impact*)) ) OR AB ( (head N2 (trauma or contact or impact*)) ) | Search modes - Find all my search terms |
| S31 | TI ( (sprain* or strain* or ruptur*) ) OR AB ( (sprain* or strain* or ruptur*) ) | Search modes - Find all my search terms |
| S32 | TI ( (fracture* or break or broke* or dislocat* or dis-locat*) ) OR AB ( (fracture* or break or broke* or dislocat* or dis-locat*) ) | Search modes - Find all my search terms |
| S33 | TI ( (laceration* or contusion* or bruis* or hematoma* or haematoma*) ) OR AB ( (laceration* or contusion* or bruis* or hematoma* or haematoma*) ) | Search modes - Find all my search terms |
| S34 | S18 OR S19 OR S20 OR S21 OR S22 OR S23 OR S24 OR S25 OR S26 OR S27 OR S28 OR S29 OR S30 OR S31 OR S32 OR S33 | Search modes - Find all my search terms |
| S35 | S3 AND S17 AND S34 | Search modes - Find all my search terms |

**SPORTDiscus with Full Text (Ebsco)**

| **#** | **Query** | **Limiters/Expanders** |
| --- | --- | --- |
| S1 | DE "RUGBY football" OR DE "COLLEGE rugby football" OR DE "FLORENTINE football" OR DE "RUGBY League football" OR DE "RUGBY Union football" OR DE "RUGBY competitions" OR DE "RUGBY football for children" OR DE "RUGBY football for girls" OR DE "SEVEN-a-side rugby football" OR DE "WOMEN'S rugby football" OR DE "RUGBY football players" OR DE "RUGBY football teams" | Search modes - Find all my search terms |
| S2 | rugby | Search modes - Find all my search terms |
| S3 | S1 OR S2 | Search modes - Find all my search terms |
| S4 | DE "TEENAGERS" OR DE "YOUTH" OR DE "CHILDREN" OR DE "HIGH school students" OR DE "YOUNG adults" | Search modes - Find all my search terms |
| S5 | DE "SCHOOLS" | Search modes - Find all my search terms |
| S6 | DE "HIGH schools" OR DE "HIGH school students" | Search modes - Find all my search terms |
| S7 | DE "MIDDLE school boys" OR DE "MIDDLE school athletes" OR DE "FEMALE middle school athletes" | Search modes - Find all my search terms |
| S8 | DE "SCHOOL children" | Search modes - Find all my search terms |
| S9 | TI ( (child* or adolescen* or youth* or teen* or juvenile* or junior or minor*) ) OR AB ( (child* or adolescen* or youth* or teen* or juvenile* or junior or minor*) ) OR KW ( (child* or adolescen* or youth* or teen* or juvenile* or junior or minor*) ) | Search modes - Find all my search terms |
| S10 | TI ( (schoolboy* or school-boy* or schoolgirl* or school-girl*) ) OR AB ( (schoolboy* or school-boy* or schoolgirl* or school-girl*) ) OR KW ( (schoolboy* or school-boy* or schoolgirl* or school-girl*) ) | Search modes - Find all my search terms |
| S11 | TI ( (boy* or girl*) ) OR AB ( (boy* or girl*) ) OR KW ( (boy* or girl*) ) | Search modes - Find all my search terms |
| S12 | TI ( (U9 or U10 or U11 or U12 or U13 or U14 or U15 or U16 or U17 or U18 or U19 or U20 or U21) ) OR AB ( (U9 or U10 or U11 or U12 or U13 or U14 or U15 or U16 or U17 or U18 or U19 or U20 or U21) ) OR KW ( (U9 or U10 or U11 or U12 or U13 or U14 or U15 or U16 or U17 or U18 or U19 or U20 or U21) ) | Search modes - Find all my search terms |
| S13 | TI ( (under N2 ("9" or "10" or "11" or "12" or "13" or "14" or "15" or "16" or "17" or "18" or "19" or "20" or "21")) ) OR AB ( (under N2 ("9" or "10" or "11" or "12" or "13" or "14" or "15" or "16" or "17" or "18" or "19" or "20" or "21")) ) OR KW ( (under N2 ("9" or "10" or "11" or "12" or "13" or "14" or "15" or "16" or "17" or "18" or "19" or "20" or "21")) ) | Search modes - Find all my search terms |
| S14 | TI ( (age* N2 ("9*" or "10*" or "11*" or "12*" or "13*" or "14*" or "15*" or "16*" or "17*" or "18*")) ) OR AB ( (age* N2 ("9*" or "10*" or "11*" or "12*" or "13*" or "14*" or "15*" or "16*" or "17*" or "18*")) ) OR KW ( (age* N2 ("9*" or "10*" or "11*" or "12*" or "13*" or "14*" or "15*" or "16*" or "17*" or "18*")) ) | Search modes - Find all my search terms |
| S15 | TI ( (("9*" or "10*" or "11*" or "12*" or "13*" or "14*" or "15*" or "16*" or "17*" or "18*") N2 (year* or yr*)) ) OR AB ( (("9*" or "10*" or "11*" or "12*" or "13*" or "14*" or "15*" or "16*" or "17*" or "18*") N2 (year* or yr*)) ) OR KW ( (("9*" or "10*" or "11*" or "12*" or "13*" or "14*" or "15*" or "16*" or "17*" or "18*") N2 (year* or yr*)) ) | Search modes - Find all my search terms |
| S16 | TI ( (young N2 (player* or athlete* or adult*)) ) OR AB ( (young N2 (player* or athlete* or adult*)) ) OR KW ( (young N2 (player* or athlete* or adult*)) ) | Search modes - Find all my search terms |
| S17 | TI ( (youth N2 (academy or player* or athlete*)) ) OR AB ( (youth N2 (academy or player* or athlete*)) ) OR KW ( (youth N2 (academy or player* or athlete*)) ) | Search modes - Find all my search terms |
| S18 | TI ( (pediatric* or paediatric*) ) OR AB ( (pediatric* or paediatric*) ) OR KW ( (pediatric* or paediatric*) ) | Search modes - Find all my search terms |
| S19 | TI school* OR AB school* OR KW school* | Search modes - Find all my search terms |
| S20 | S4 OR S5 OR S6 OR S7 OR S8 OR S9 OR S10 OR S11 OR S12 OR S13 OR S14 OR S15 OR S16 OR S17 OR S18 OR S19 | Search modes - Find all my search terms |
| S21 | DE "RUGBY football injuries" | Search modes - Find all my search terms |
| S22 | DE "SPORTS injuries" | Search modes - Find all my search terms |
| S23 | DE "SPRAINS" | Search modes - Find all my search terms |
| S24 | DE "SOFT tissue injuries" OR DE "LIGAMENT injuries" OR DE "TENDON injuries" | Search modes - Find all my search terms |
| S25 | DE "ROTATOR cuff injuries" | Search modes - Find all my search terms |
| S26 | DE "BRAIN concussion" OR DE "BRAIN injuries" | Search modes - Find all my search terms |
| S27 | DE "HEAD injuries" | Search modes - Find all my search terms |
| S28 | DE "BONE fractures" OR DE "ANKLE fractures" OR DE "AVULSION fractures" OR DE "CARTILAGE fractures" OR DE "COMMINUTED fractures" OR DE "FACIAL bone fractures" OR DE "FRACTURE healing" OR DE "HEEL bone fractures" OR DE "JONES fracture" OR DE "PELVIC fractures" OR DE "STRESS fractures (Orthopedics)" OR DE "WRIST fractures" | Search modes - Find all my search terms |
| S29 | DE "FACIAL injuries" | Search modes - Find all my search terms |
| S30 | DE "NECK injuries" | Search modes - Find all my search terms |
| S31 | DE "ARM injuries" OR DE "HAND injuries" | Search modes - Find all my search terms |
| S32 | DE "BACK injuries" | Search modes - Find all my search terms |
| S33 | DE "LEG injuries" OR DE "ANKLE injuries" OR DE "FOOT injuries" OR DE "TIBIA injuries" | Search modes - Find all my search terms |
| S34 | DE "BRUISES" | Search modes - Find all my search terms |
| S35 | DE "JOINT dislocations" OR DE "ANKLE dislocation" OR DE "ELBOW dislocation" OR DE "FINGER dislocation" OR DE "WRIST dislocations" OR DE "SUBLUXATION" | Search modes - Find all my search terms |
| S36 | DE "LIGAMENT injuries" OR DE "COLLATERAL ligament injuries" OR DE "CRUCIATE ligament injuries" | Search modes - Find all my search terms |
| S37 | DE "KNEE injuries" OR DE "ANTERIOR cruciate ligament injuries" OR DE "PATELLAR ligament injuries" OR DE "POSTERIOR cruciate ligament injuries" | Search modes - Find all my search terms |
| S38 | TI injur* OR AB injur* OR KW injur* | Search modes - Find all my search terms |
| S39 | TI ( (concuss* or mtbi*) ) OR AB ( (concuss* or mtbi*) ) OR KW ( (concuss* or mtbi*) ) | Search modes - Find all my search terms |
| S40 | TI ( (head N2 (trauma or contact or impact*)) ) OR AB ( (head N2 (trauma or contact or impact*)) ) OR KW ( (head N2 (trauma or contact or impact*)) ) | Search modes - Find all my search terms |
| S41 | TI ( (sprain* or strain* or ruptur*) ) OR AB ( (sprain* or strain* or ruptur*) ) OR KW ( (sprain* or strain* or ruptur*) ) | Search modes - Find all my search terms |
| S42 | TI ( (fracture* or break or broke* or dislocat* or dis-locat*) ) OR AB ( (fracture* or break or broke* or dislocat* or dis-locat*) ) OR KW ( (fracture* or break or broke* or dislocat* or dis-locat*) ) | Search modes - Find all my search terms |
| S43 | TI ( (laceration* or contusion* or bruis* or hematoma* or haematoma*) ) OR AB ( (laceration* or contusion* or bruis* or hematoma* or haematoma*) ) OR KW ( (laceration* or contusion* or bruis* or hematoma* or haematoma*) ) | Search modes - Find all my search terms |
| S44 | S21 OR S22 OR S23 OR S24 OR S25 OR S26 OR S27 OR S28 OR S29 OR S30 OR S31 OR S32 OR S33 OR S34 OR S35 OR S36 OR S37 OR S38 OR S39 OR S40 OR S41 OR S42 OR S43 | Search modes - Find all my search terms |
| S45 | S3 AND S20 AND S44 | Search modes - Find all my search terms |

**ERIC (Ebsco)**

| **#** | **Query** | **Limiters/Expanders** |
| --- | --- | --- |
| S1 | rugby | Search modes - Find all my search terms |
| S2 | DE "Adolescents" OR DE "Children" OR DE "Early Adolescents" OR DE "High School Students" OR DE "Late Adolescents" OR DE "Preadolescents" OR DE "Secondary School Students" OR DE "Youth" | Search modes - Find all my search terms |
| S3 | DE "Schools" OR DE "Elementary Schools" OR DE "Middle Schools" | Search modes - Find all my search terms |
| S4 | TI ( (child* or adolescen* or youth* or teen* or juvenile* or junior or minor*) ) OR AB ( (child* or adolescen* or youth* or teen* or juvenile* or junior or minor*) ) OR KW ( (child* or adolescen* or youth* or teen* or juvenile* or junior or minor*) ) | Search modes - Find all my search terms |
| S5 | TI ( (schoolboy* or school-boy* or schoolgirl* or school-girl*) ) OR AB ( (schoolboy* or school-boy* or schoolgirl* or school-girl*) ) OR KW ( (schoolboy* or school-boy* or schoolgirl* or school-girl*) ) | Search modes - Find all my search terms |
| S6 | TI ( (boy* or girl*) ) OR AB ( (boy* or girl*) ) OR KW ( (boy* or girl*) ) | Search modes - Find all my search terms |
| S7 | TI ( (U9 or U10 or U11 or U12 or U13 or U14 or U15 or U16 or U17 or U18 or U19 or U20 or U21) ) OR AB ( (U9 or U10 or U11 or U12 or U13 or U14 or U15 or U16 or U17 or U18 or U19 or U20 or U21) ) OR KW ( (U9 or U10 or U11 or U12 or U13 or U14 or U15 or U16 or U17 or U18 or U19 or U20 or U21) ) | Search modes - Find all my search terms |
| S8 | TI ( (under N2 ("9" or "10" or "11" or "12" or "13" or "14" or "15" or "16" or "17" or "18" or "19" or "20" or "21")) ) OR AB ( (under N2 ("9" or "10" or "11" or "12" or "13" or "14" or "15" or "16" or "17" or "18" or "19" or "20" or "21")) ) OR KW ( (under N2 ("9" or "10" or "11" or "12" or "13" or "14" or "15" or "16" or "17" or "18" or "19" or "20" or "21")) ) | Search modes - Find all my search terms |
| S9 | TI ( (age* N2 ("9*" or "10*" or "11*" or "12*" or "13*" or "14*" or "15*" or "16*" or "17*" or "18*")) ) OR AB ( (age* N2 ("9*" or "10*" or "11*" or "12*" or "13*" or "14*" or "15*" or "16*" or "17*" or "18*")) ) OR KW ( (age* N2 ("9*" or "10*" or "11*" or "12*" or "13*" or "14*" or "15*" or "16*" or "17*" or "18*")) ) | Search modes - Find all my search terms |
| S10 | TI ( (("9*" or "10*" or "11*" or "12*" or "13*" or "14*" or "15*" or "16*" or "17*" or "18*") N2 (year* or yr*)) ) OR AB ( (("9*" or "10*" or "11*" or "12*" or "13*" or "14*" or "15*" or "16*" or "17*" or "18*") N2 (year* or yr*)) ) OR KW ( (("9*" or "10*" or "11*" or "12*" or "13*" or "14*" or "15*" or "16*" or "17*" or "18*") N2 (year* or yr*)) ) | Search modes - Find all my search terms |
| S11 | TI ( (young N2 (player* or athlete* or adult*)) ) OR AB ( (young N2 (player* or athlete* or adult*)) ) OR KW ( (young N2 (player* or athlete* or adult*)) ) | Search modes - Find all my search terms |
| S12 | TI ( (youth N2 (academy or player* or athlete*)) ) OR AB ( (youth N2 (academy or player* or athlete*)) ) OR KW ( (youth N2 (academy or player* or athlete*)) ) | Search modes - Find all my search terms |
| S13 | TI ( (pediatric* or paediatric*) ) OR AB ( (pediatric* or paediatric*) ) OR KW ( (pediatric* or paediatric*) ) | Search modes - Find all my search terms |
| S14 | TI school* OR AB school* OR KW school* | Search modes - Find all my search terms |
| S15 | DE "Head Injuries" OR DE "Injuries" | Search modes - Find all my search terms |
| S16 | TI injur* OR AB injur* OR KW injur* | Search modes - Find all my search terms |
| S17 | TI ( (concuss* or mtbi*) ) OR AB ( (concuss* or mtbi*) ) OR KW ( (concuss* or mtbi*) ) | Search modes - Find all my search terms |
| S18 | TI ( (head N2 (trauma or contact or impact*)) ) OR AB ( (head N2 (trauma or contact or impact*)) ) OR KW ( (head N2 (trauma or contact or impact*)) ) | Search modes - Find all my search terms |
| S19 | TI ( (sprain* or strain* or ruptur*) ) OR AB ( (sprain* or strain* or ruptur*) ) OR KW ( (sprain* or strain* or ruptur*) ) | Search modes - Find all my search terms |
| S20 | TI ( (fracture* or break or broke* or dislocat* or dis-locat*) ) OR AB ( (fracture* or break or broke* or dislocat* or dis-locat*) ) OR KW ( (fracture* or break or broke* or dislocat* or dis-locat*) ) | Search modes - Find all my search terms |
| S21 | TI ( (laceration* or contusion* or bruis* or hematoma* or haematoma*) ) OR AB ( (laceration* or contusion* or bruis* or hematoma* or haematoma*) ) OR KW ( (laceration* or contusion* or bruis* or hematoma* or haematoma*) ) | Search modes - Find all my search terms |
| S22 | S2 OR S3 OR S4 OR S5 OR S6 OR S7 OR S8 OR S9 OR S10 OR S11 OR S12 OR S13 OR S14 | Search modes - Find all my search terms |
| S23 | S15 OR S16 OR S17 OR S18 OR S19 OR S20 OR S21 | Search modes - Find all my search terms |
| S24 | S1 AND S22 AND S23 | Search modes - Find all my search terms |

**Web of Science. Core Collection**Including: Science Citation Index-Expanded, Social Sciences Citation Index, Arts & Humanities Citation Index, Conference Proceedings Citation Index - Science, Conference Proceedings Citation Index - Social Sciences & Humanities, and Emerging Sources Citation Index


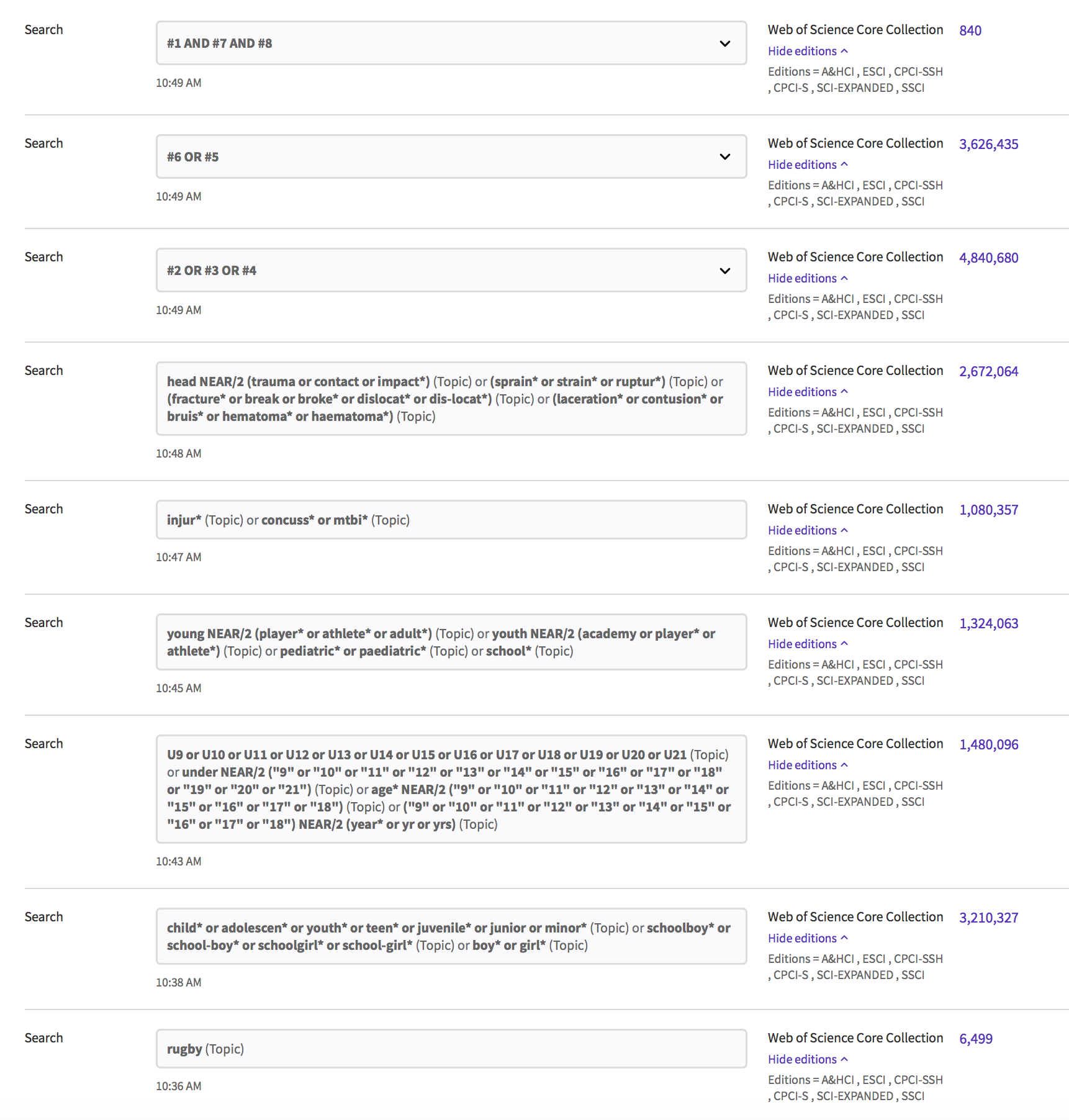


Appendix 2: PRISMA abstract checklist

| **Section and Topic** | **Item #** | **Checklist item** | **Reported (Yes/No)** |
| --- | --- | --- | --- |
| **TITLE** | | |  |
| Title | 1 | Identify the report as a systematic review. | Yes |
| **BACKGROUND** | | |  |
| Objectives | 2 | Provide an explicit statement of the main objective(s) or question(s) the review addresses. | Yes |
| **METHODS** | | |  |
| Eligibility criteria | 3 | Specify the inclusion and exclusion criteria for the review. | Yes |
| Information sources | 4 | Specify the information sources (e.g. databases, registers) used to identify studies and the date when each was last searched. | Yes- reference to propsero. |
| Risk of bias | 5 | Specify the methods used to assess risk of bias in the included studies. | Yes |
| Synthesis of results | 6 | Specify the methods used to present and synthesise results. | Yes |
| **RESULTS** | | |  |
| Included studies | 7 | Give the total number of included studies and participants and summarise relevant characteristics of studies. | Yes- Number of participants not available for all studies. |
| Synthesis of results | 8 | Present results for main outcomes, preferably indicating the number of included studies and participants for each. If meta-analysis was done, report the summary estimate and confidence/credible interval. If comparing groups, indicate the direction of the effect (i.e. which group is favoured). | Yes |
| **DISCUSSION** | | |  |
| Limitations of evidence | 9 | Provide a brief summary of the limitations of the evidence included in the review (e.g. study risk of bias, inconsistency and imprecision). | Yes |
| Interpretation | 10 | Provide a general interpretation of the results and important implications. | Yes |
| **OTHER** | | |  |
| Funding | 11 | Specify the primary source of funding for the review. | Yes- on title page |
| Registration | 12 | Provide the register name and registration number. | Yes |

Appendix 3: PRISMA Checklist

| **Section and Topic** | **Item #** | **Checklist item** | **Location where item is reported** |
| --- | --- | --- | --- |
| **TITLE** | | |  |
| Title | 1 | Identify the report as a systematic review. | P1 L1 |
| **ABSTRACT** | | |  |
| Abstract | 2 | See the PRISMA 2020 for Abstracts checklist. | Separate PRISMA form |
| **INTRODUCTION** | | |  |
| Rationale | 3 | Describe the rationale for the review in the context of existing knowledge. | P1 L5- L19 |
| Objectives | 4 | Provide an explicit statement of the objective(s) or question(s) the review addresses. | P1 L19-21 |
| **METHODS** | | |  |
| Eligibility criteria | 5 | Specify the inclusion and exclusion criteria for the review and how studies were grouped for the syntheses. | P2 L13 –24 |
| Information sources | 6 | Specify all databases, registers, websites, organisations, reference lists and other sources searched or consulted to identify studies. Specify the date when each source was last searched or consulted. | P1 L29 – P2 L1 |
| Search strategy | 7 | Present the full search strategies for all databases, registers and websites, including any filters and limits used. | Appendix 1 |
| Selection process | 8 | Specify the methods used to decide whether a study met the inclusion criteria of the review, including how many reviewers screened each record and each report retrieved, whether they worked independently, and if applicable, details of automation tools used in the process. | P2 L9 – 13 |
| Data collection process | 9 | Specify the methods used to collect data from reports, including how many reviewers collected data from each report, whether they worked independently, any processes for obtaining or confirming data from study investigators, and if applicable, details of automation tools used in the process. | P2 L28-33 |
| Data items | 10a | List and define all outcomes for which data were sought. Specify whether all results that were compatible with each outcome domain in each study were sought (e.g. for all measures, time points, analyses), and if not, the methods used to decide which results to collect. | P2 L33- P3 L4 |
|  | 10b | List and define all other variables for which data were sought (e.g. participant and intervention characteristics, funding sources). Describe any assumptions made about any missing or unclear information. | P2 L33- P3 L4 |
| Study risk of bias assessment | 11 | Specify the methods used to assess risk of bias in the included studies, including details of the tool(s) used, how many reviewers assessed each study and whether they worked independently, and if applicable, details of automation tools used in the process. | P3 L4 – L6 |
| Effect measures | 12 | Specify for each outcome the effect measure(s) (e.g. risk ratio, mean difference) used in the synthesis or presentation of results. | P3 L9-13, P3 L29 – 31,  P4 L6 – 8, |
| Synthesis methods | 13a | Describe the processes used to decide which studies were eligible for each synthesis (e.g. tabulating the study intervention characteristics and comparing against the planned groups for each synthesis (item #5)). | P3 L9- P4 L25  Figure 1, P4 L2-9 |
|  | 13b | Describe any methods required to prepare the data for presentation or synthesis, such as handling of missing summary statistics, or data conversions. | P3 L15- 27 |
|  | 13c | Describe any methods used to tabulate or visually display results of individual studies and syntheses. | P6 L13 -15. |
|  | 13d | Describe any methods used to synthesize results and provide a rationale for the choice(s). If meta-analysis was performed, describe the model(s), method(s) to identify the presence and extent of statistical heterogeneity, and software package(s) used. | P3 L9-27 |
|  | 13e | Describe any methods used to explore possible causes of heterogeneity among study results (e.g. subgroup analysis, meta-regression). | P3 2-3, P6 L 26- P7 L2 |
|  | 13f | Describe any sensitivity analyses conducted to assess robustness of the synthesized results. | P6 L17 - 25 |
| Reporting bias assessment | 14 | Describe any methods used to assess risk of bias due to missing results in a synthesis (arising from reporting biases). | P3 L4-6 P6 19-25 P4 L22-26 |
| Certainty assessment | 15 | Describe any methods used to assess certainty (or confidence) in the body of evidence for an outcome. | P3 L13-15  P3 L30-32 |
| **RESULTS** | | |  |
| Study selection | 16a | Describe the results of the search and selection process, from the number of records identified in the search to the number of studies included in the review, ideally using a flow diagram. | Figure 1 |
|  | 16b | Cite studies that might appear to meet the inclusion criteria, but which were excluded, and explain why they were excluded. | P20 L15 – 18 |
| Study characteristics | 17 | Cite each included study and present its characteristics. | Table S1 |
| Risk of bias in studies | 18 | Present assessments of risk of bias for each included study. | Table S1 |
| Results of individual studies | 19 | For all outcomes, present, for each study: (a) summary statistics for each group (where appropriate) and (b) an effect estimate and its precision (e.g. confidence/credible interval), ideally using structured tables or plots. | Figure 2,3, 4,5,6 Table 1,2,3, 4, S2, S3, S8, S9, S10, S11, S12 S13, S14, S15 |
| Results of syntheses | 20a | For each synthesis, briefly summarise the characteristics and risk of bias among contributing studies. | P3 L9-16  Figure S1 |
|  | 20b | Present results of all statistical syntheses conducted. If meta-analysis was done, present for each the summary estimate and its precision (e.g. confidence/credible interval) and measures of statistical heterogeneity. If comparing groups, describe the direction of the effect. | Figure 2,3, 4,5,6 Table 1,2,3, 4, S2, S3, S8, S9, S10, S11, S12 S13, S14, S15 |
|  | 20c | Present results of all investigations of possible causes of heterogeneity among study results. | P3 L9-15  P4, L5- 6 |
|  | 20d | Present results of all sensitivity analyses conducted to assess the robustness of the synthesized results. | P6 L17-25 |
| Reporting biases | 21 | Present assessments of risk of bias due to missing results (arising from reporting biases) for each synthesis assessed. | P6 L17-25 |
| Certainty of evidence | 22 | Present assessments of certainty (or confidence) in the body of evidence for each outcome assessed. | P3 L13-15  P3 L31  Figure 2,3, 4,5,6 Table 1,2,3, 4, S2, S3, S8, S9, S10, S11, S12 S13, S14, S15 |
| **DISCUSSION** | | |  |
| Discussion | 23a | Provide a general interpretation of the results in the context of other evidence. | P19 L22-24  P20 L32- P21 L2 |
|  | 23b | Discuss any limitations of the evidence included in the review. | P22 L19-35 |
|  | 23c | Discuss any limitations of the review processes used. | P22 L19-35 |
|  | 23d | Discuss implications of the results for practice, policy, and future research. | P23 L2-14 |
| **OTHER INFORMATION** | | |  |
| Registration and protocol | 24a | Provide registration information for the review, including register name and registration number, or state that the review was not registered. | P1 L25 |
|  | 24b | Indicate where the review protocol can be accessed, or state that a protocol was not prepared. | P1 L25 |
|  | 24c | Describe and explain any amendments to information provided at registration or in the protocol. | P2 L16-19. |
| Support | 25 | Describe sources of financial or non-financial support for the review, and the role of the funders or sponsors in the review. | Title Page- Competing interests |
| Competing interests | 26 | Declare any competing interests of review authors. | Title Page- Competing interests |
| Availability of data, code and other materials | 27 | Report which of the following are publicly available and where they can be found: template data collection forms; data extracted from included studies; data used for all analyses; analytic code; any other materials used in the review. | Appendix 1 |
